# ERK5 Is a Major Determinant of Chemical Sarcomagenesis: Implications in Human Pathology

**DOI:** 10.3390/cancers14143509

**Published:** 2022-07-19

**Authors:** Elena Arconada-Luque, Jaime Jiménez-Suarez, Raquel Pascual-Serra, Syong Hyun Nam-Cha, Teresa Moline, Francisco J. Cimas, Germán Fliquete, Marta Ortega-Muelas, Olga Roche, Diego M. Fernández-Aroca, Raúl Muñoz Velasco, Natalia García-Flores, Cristina Garnés-García, Adrián Sánchez-Fdez, Sofía Matilla-Almazán, Víctor J. Sánchez-Arévalo Lobo, Javier Hernández-Losa, Borja Belandia, Atanasio Pandiella, Azucena Esparís-Ogando, Santiago Ramón y Cajal, Luis del Peso, Ricardo Sánchez-Prieto, María José Ruiz-Hidalgo

**Affiliations:** 1 Laboratorio de Oncología Molecular, Unidad de Medicina Molecular, Centro Regional de Investigaciones Biomédicas, Unidad Asociada de Biomedicina UCLM, Unidad Asociada al CSIC, Universidad de Castilla-La Mancha, 02008 Albacete, Spain; elena.arconada@alu.uclm.es (E.A.-L.); jaime.jimenez@uclm.es (J.J.-S.); rpascuals@sescam.jccm.es (R.P.-S.); marta.ortega.muelas@gmail.com (M.O.-M.); oroche@gmail.com (O.R.); diego.fernandez@uclm.es (D.M.F.-A.); natalia.garcia16@alu.uclm.es (N.G.-F.); cristina.garnes@alu.uclm.es (C.G.-G.); maria.rhidalgo@uclm.es (M.J.R.-H.); 2 Servicio de Anatomía Patológica, Hospital General de Albacete, 02008 Albacete, Spain; shnam@sescam.jccm.es; 3 Grupo de Patología Molecular Traslacional, Vall d’Hebron Institut de Recerca, Universitat Autònoma de Barcelona Centro de Investigación Biomédica en RED de Cancer CIBERONC, 08035 Barcelona, Spain; teresa.moline@vhir.org (T.M.); gfliquete@vhebron.net (G.F.); jahernan@vhebron.net (J.H.-L.); sramon@vhebron.net (S.R.y.C.); 4 Unidad de Bioquímica y Biología Molecular, Servicio de Instrumentación Biomédica, Universidad de Castilla-La Mancha, 02008 Albacete, Spain; franciscojose.cimas@uclm.es; 5 Departamento de Ciencias Médicas, Facultad de Medicina, Universidad de Castilla-La Mancha, 02008 Albacete, Spain; 6 Grupo de Oncología Molecular, Facultad de Ciencias Experimentales, Instituto de Investigación Biosanitaria, Universidad Francisco de Vitoria, Pozuelo de Alarcón, 28223 Madrid, Spain; raul.munoz@ufv.es (R.M.V.); victor.sanchezarevalo@ufv.es (V.J.S.-A.L.); 7 Departamento de Anatomía Patológica, Instituto de Investigación Hospital 12 de Octubre, Av. Córdoba, s/n, 28041 Madrid, Spain; 8 Instituto de Biología Molecular y Celular del Cáncer-CSIC, 37007 Salamanca, Spain; adriansf@usal.es (A.S.-F.); sofiamatilla@gmail.com (S.M.-A.); atanasio@usal.es (A.P.); esparis@usal.es (A.E.-O.); 9 Instituto de Investigación Biomédica de Salamanca (IBSAL), Hospital Universitario de Salamanca, Universidad de Salamanca, CSIC, 37007 Salamanca, Spain; 10 Centro de Investigación Biomédica en RED de Cancer CIBERONC, 37007 Salamanca, Spain; 11 Departamento de Biología del Cáncer, Instituto de Investigaciones Biomédicas ‘Alberto Sols’ (CSIC-UAM), Unidad Asociada de Biomedicina UCLM, Unidad Asociada al CSIC, 28029 Madrid, Spain; bbelandia@iib.uam.es; 12 Departamento de Bioquímica, Universidad Autónoma de Madrid (UAM) and Instituto de Investigaciones Biomédicas ‘Alberto Sols’ (CSIC-UAM), 28029 Madrid, Spain; lpeso@uam.es; 13 Unidad Asociada de Biomedicina UCLM, Unidad Asociada al CSIC, 28029 Madrid, Spain; 14 Centro de Investigación Biomédica en Red de Enfermedades Respiratorias CIBERES, 28029 Madrid, Spain; 15 Instituto de Investigaciones Biomédicas ‘Alberto Sols’, Consejo Superior de Investigaciones Científicas (IIBM-CSIC)-Universidad de Castilla-La Mancha, 02008 Albacete, Spain; 16 Departamento de Química Inorgánica, Orgánica y Bioquímica, Área de Bioquímica y Biología Molecular, Facultad de Medicina, Universidad de Castilla-La Mancha, 02008 Albacete, Spain

**Keywords:** ERK5, *MAPK7*, soft tissue sarcoma, leiomyosarcoma, rhabdomyosarcoma, KLF2, 3-methyl-cholanthrene

## Abstract

**Simple Summary:**

Sarcoma is a heterogeneous group of tumors poorly studied with few therapeutic opportunities. Interestingly, the role of MAPKs still remains unclear in sarcomatous pathology. Here, we describe for the first time the critical role of ERK5 in the biology of soft tissue sarcoma by using in vitro and in vivo approaches in a murine experimental model of chemical sarcomagenesis. Indeed, our observations were extrapolated to a short series of human leiomyosarcoma and rhabdomyosarcomas. Furthermore, transcriptome analysis allows us to demonstrate the critical role of KLF2 in the biological effects of ERK5. Therefore, the data presented here open new windows in the diagnosis and therapy of soft tissue sarcomas.

**Abstract:**

Sarcomas are a heterogeneous group of tumors in which the role of ERK5 is poorly studied. To clarify the role of this MAPK in sarcomatous pathology, we used a murine 3-methyl-cholanthrene (3MC)-induced sarcoma model. Our data show that 3MC induces pleomorphic sarcomas with muscle differentiation, showing an increased expression of ERK5. Indeed, this upregulation was also observed in human sarcomas of muscular origin, such as leiomyosarcoma or rhabdomyosarcoma. Moreover, in cell lines derived from these 3MC-induced tumors, abrogation of *Mapk7* expression by using specific shRNAs decreased in vitro growth and colony-forming capacity and led to a marked loss of tumor growth in vivo. In fact, transcriptomic profiling in ERK5 abrogated cell lines by RNAseq showed a deregulated gene expression pattern for key biological processes such as angiogenesis, migration, motility, etc., correlating with a better prognostic in human pathology. Finally, among the various differentially expressed genes, *Klf2* is a key mediator of the biological effects of ERK5 as indicated by its specific interference, demonstrating that the ERK5–KLF2 axis is an important determinant of sarcoma biology that should be further studied in human pathology.

## 1. Introduction

Sarcomas are a group of heterogeneous tumors that develop from the connective tissue, which provides a supporting matrix throughout the organism. More than 150 types and subtypes of sarcomas have been described. The classification of sarcomas divides them into soft tissue sarcomas (STS) and bone sarcomas. In addition, there is a third group, gastrointestinal stromal tumors, which are soft tissue sarcomas considered a separate group due to their diagnostic and therapeutic characteristics (for a review, see [1]). Sarcomas are rare tumors that, in adults, comprise 1% of all cancers; however, in children, they represent about 15% of cancer cases. In 2022, it is estimated that more than 13,000 new cases of STS will be diagnosed in the USA, and more than 5000 people will die of this disease (ASCO, https://www.cancer.net/, accessed on 1 April 2022, ACS www.cancer.org, accessed on 1 April 2022). Therapeutically, the most common treatments are surgery and chemo/radiotherapy. However, the use of novel anti-tumor agents such as kinase inhibitors or immunotherapy is now being considered (for a review, see [2]).

Currently, there are different animal models to study STS [3,4,5], which have revealed the implication of key proteins in sarcomagenesis, as in the case of p53 [6] or the AKT signaling pathway [7,8]. Indeed, most of this experimental observation has been extrapolated to human pathologies, which will certainly have clinical implications in the coming years. A method that has been widely used for the induction of sarcomas since the first half of the last century is the use of carcinogens, being 3-methyl-cholanthrene (3MC) a great example [9]. The tumor developed after intramuscular injection of this carcinogen is qualified as “sarcoma”, and it has been used as a basis for demonstrating the oncogenic/tumor suppressor properties of a multitude of proteins, including cohesin SA1, p53, ATM, and PIM kinases, among others [10,11,12]. Furthermore, the model of 3MC-derived sarcoma has been characterized by WES, showing specific alterations in oncogenes and tumor suppressors such as *K-ras* or *Tp53* [13], also implicated in human tumors as leiomyosarcoma or rhabdomyosarcoma [14]. However, it is notorious that a definitive pathological characterization has not yet been proposed for this experimental model.

Interestingly, MAPK signaling has been related to several types of sarcomas [15,16], and recent evidence supports that modulation of MAPKs could have therapeutic implications [17,18,19]. However, it is remarkable that there is no work detailing the role of MAPKs in the sarcoma model of 3MC. In fact, ERK5, one of the canonical members of MAPKs superfamily described more than 25 years ago [20,21], is highly expressed in sarcomatous pathology, as indicated in The Cancer Genome Atlas (TCGA) series (see below). Interestingly, this particular MAPK signaling pathway has been linked to cancer [22,23], and it is expected to exploit its clinical implications in the coming years [24]. Indeed, ERK5 has been related to several types of tumors such as breast [25], multiple myeloma [26], or cholangiocarcinoma [27]. However, no deep studies evaluating the role of the ERK5 signaling pathway in STS have been performed to date.

Here, we describe how the ERK5 pathway, with the direct implication of KLF2, is necessary for chemical sarcomagenesis triggered by 3MC, suggesting that this signaling axis needs to be deeply studied in order to fully exploit its potential in diagnostic and therapy in human sarcomatous pathology.

## 2. Materials and Methods

### 2.1. Cell Lines

HEK293T and C3H10t1/2 cells were purchased from ATCC (LGC, Barcelona, Spain). Cells were maintained in 5% CO_2_ at 37 °C and grown in Dulbecco’s modified Eagle’s medium (DMEM) supplemented with 10% fetal bovine serum (FBS), 1% glutamine plus antibiotics. All cell culture reagents were provided by Lonza (Culteck, Madrid, Spain).

### 2.2. Antibodies and Chemicals

The antibodies used are listed in Supplementary Table S1. 3MC, etoposide, and crystal violet were purchased from Merck (Madrid, Spain). XMD8-92 was obtained from Selleckchem (Madrid, Spain).

### 2.3. Animal Studies

All the animal experimentation was carried out according to the NIH-Intramural Animal Guide for the Care and Use of Laboratory Animals and approved by the Ethics in Animal Care Committee of the University of Castilla-La Mancha (reference PR-2019-07-18).

### 2.4. Induction of Murine Sarcoma and Cell Lines Derivation

C57BL/6 females (n = 4) were injected in the right hind with 3MC as previously described [28]. When the tumors reached a size of 1–1.5 cm in diameter, mice were euthanized by cervical dislocation. Cell lines were derived from the tumors as previously described [28]. In this study, two cell lines out of four (referred to as 3MC-C1 and 3MC-C3) were chosen as a representative model of murine sarcoma. Cells were maintained in 5% CO_2_ at 37 °C and grown in DMEM supplemented with 10% FBS, 1% glutamine plus antibiotics.

### 2.5. Xenograft Assays

For xenograft assays, 5 × 10^5^ cells from 3MC-C1, 3MC-C3, or derived cell lines were subcutaneously injected into the back of 5/6-week-old female mice of J:NMRIFoxn1nu/Foxn1nu strain (Janvier). Tumors were measured by caliper every two days, and tumor volume was calculated according to the formula V = (D × d^2^)/2 (where D is tumor length and d tumor width). Figures show an experiment out of 2 performed with 2 different pools of infection that rendered nearly identical results.

### 2.6. RNA Interference Assays, Lentiviral Production, and Infections

Lentiviral production and cell infection were performed as previously described [28] by using the following shRNAs from Merck (Madrid, Spain): PLKO.1-shRNA ERK5-1 (TRC-N-0000232396), PLKO.1-shRNA ERK5-2 (TRC-N-0000232397), PLKO.1-shRNA KLF2 (TRC-N-0000295770) or PLKO.1-empty vector (SHC001). Forty-eight hours post-infection, cells were selected with puromycin (2.5 µg/mL Merck) for 72 h. Each experiment was performed with at least two different pools of infection. Infected cells were discarded 15–20 days after selection, and new pools were generated.

### 2.7. Western Blotting

Protein quantification and western blot were performed as previously described [29]. For tumor samples, 400 μL of lysis buffer was added to 0.2 g of tumor, and it was disaggregated using a Polytron PT-2100 (Kinematica AG, Malters, Switzerland). Once the tissue was homogenized, the above-mentioned protocol was followed.

### 2.8. Clonogenic Assay

Clonogenic assays were performed as previously described [28] by using 200 cells/well seeded in 6-well plates and maintained for 12–14 days. The colonies with less than 5 mm diameter were discarded.

### 2.9. Growth Curves

Growth curves were performed as previously described [30]. Briefly, 3 × 10^5^ cells for 3MC-C1 and 5 × 10^5^ cells for 3MC-C3 were seeded into 100 mm plates and counted on days 3, 6, and 9 by using an automated cell counter (Bio-Rad) and replated in the same manner up to day 9. This experiment was performed with 3 different pools of infection for each cell line.

### 2.10. Adhesion Assay

For adhesion assays, 24-well plates were coated with fibronectin (10 μg/mL in PBS, 1 h, 37 °C, Merck). Then, fibronectin was removed, and plates were blocked (0.5% BSA in DMEM, 45 min) and washed (0.1% BSA in DMEM). After cold shock, 1 × 10^5^ cells were seeded. The medium was removed at different time points, and adhered cells were washed 3× with PBS. Cells were stained with crystal violet for 15 min, washed with deionized water, and dried at RT for 24 h. Then, crystal violet was dissolved in acetic acid (10% in deionized water, 15 min on mild shaking), and absorbance was measured at 590 nm using a Biokinetics plate reader.

### 2.11. RNA Isolation, Reverse Transcription, and Real-Time Quantitative PCR (RT-qPCR)

Total RNA from cell and mice tumor samples (after tissue homogenization with a Polytron) was obtained as previously described [28]. For samples embedded in paraffin, total RNA was isolated using the Roche High Pure FFPET RNA Isolation Kit (06650775001, (Merck Madrid, Spain) following the manufacturer’s protocol. cDNA synthesis and PCR conditions were performed as previously described [28]. Primers were designed by using the NCBI BLAST software and purchased from Sigma-Aldrich. The primers used are listed in Supplementary Table S2.

### 2.12. Histology and Immunohistochemistry

Histology and immunohistochemistry assays were performed as previously described [15]. Human tumor samples were provided by the Tumor Banks of the Vall d’Hebron University Hospital and the General Hospital of Albacete with appropriate ethical approval (number 2019/07/071).

### 2.13. RNA Sequencing

Cells infected with PLKO.1-empty vector or PLKO.1-shRNA ERK5-1 were grown up to 90% confluency after selection, split 1/3, and allowed to grow again. Two different pools for 3MC-C1 and three different pools for 3MC-C3 were used. For each pool of infection, new viruses were produced with at least 2 weeks of difference. Total RNA was extracted, and RNA integrity was determined by Agilent 2100 Bioanalyzer (RIN range, 9.1–9.9). cDNA libraries were generated from 600 ng of RNA from each sample using the Illumina TruSeq Stranded mRNA Sample Preparation kit (Illumina, San Diego, CA, USA). Libraries were sequenced on an Ilumina HiSeq2000 apparatus according to the manufacturer’s protocols, and a minimum of 9 million 100-base single-reads were generated from each sample.

Pseudocounts for each gene were obtained from sequencing reads with salmon software [31] using RefSeq [32] mRNA sequences for mouse genome assembly GRCm38/mm10 as reference. Differential expression in individual subsets was calculated with the R package DESeq2 [33] using default settings. RNAseq data are publicly available from the NCBI’s GEO repository with accession number GSE199395.

### 2.14. Functional Enrichment Analysis

Enrichment of Gene Ontology terms was performed with the Bioconductor’s clusterProfiler package [34] using a q cut-off value of 0.05.

### 2.15. In Silico Analysis

MAPKs expression in TGCA series was analyzed by using the Timer2.0 website. The top 30 up-regulated and down-regulated gene signature after *Mapk7* abrogation was identified in terms of log2 of Fold Change, and prognosis correlation with expression levels was evaluated. To associate the level of gene expression with prognosis, the median expression values for different transcripts were used as a cut-off to discriminate “high” and “low” expression cohorts, which are compared using a Cox survival analysis (proportional hazards). Graphics were drawn using the ggplot2 package running in R Studio Version 1.2.5033. For ERK5 protein-protein interaction network analysis, the STRING website (https://www.string-db.org/cgi/input?sessionId=bfLuXEqaflvN&input_page_show_search=off, accessed on 3 April 2022) was used.

### 2.16. Statistical Analysis

The data are reported as the mean ± standard deviation (SD). Statistical analysis was performed using the GraphPadPrism 9 and Office Excel 2020 (Microsoft). Significance was determined using a *t*-test and a two-way ANOVA test. The statistical significance of differences is indicated in Figures by asterisks as follows: * *p* < 0.05; ** *p* < 0.01; and *** *p* < 0.001.

## 3. Results

### 3.1. ERK5 Is Upregulated in 3MC-Induced Sarcoma and in Human Leiomyosarcoma

**Figure 1:**
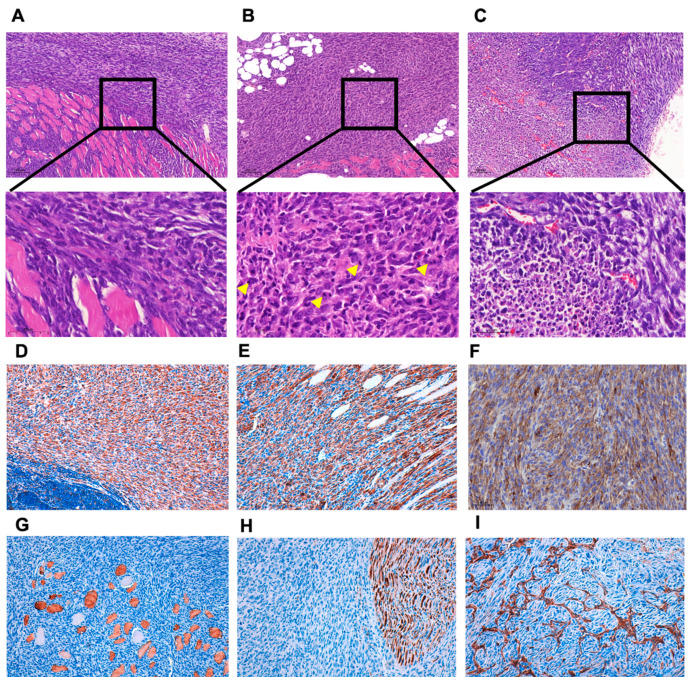
**Histological and immunohistochemical characterization of 3MC-induced tumors**. Representative hematoxylin and eosin-stained fixed sections of tumors induced by 3MC. (**A**) At low magnification (10×), the tumors show high cellular density and infiltrated local muscle fibers. (**B**) At high magnification (40×), the cells display spindle or epithelial morphology, nuclear atypia, and frequent mitosis (indicated by yellow arrows). (**C**) There are large areas of necrotic cells located at the center of the tumor. Original magnification is 10×; insets are shown at a 40× magnification. Representative images of immunohistochemical staining in fixed section of tumors induced by 3MC. The tumor cells were positive for vimentin (**D**), smooth muscle actin (**E**), and caldesmon (**F**). Desmin (**G**), protein S100 (**H**), and CD34 (**I**) were negative. Pictures are shown at a 20× magnification.

After 120 days of the 3MC injection in the left rear, mice developed a tumor mass between 1 and 1.5 cm in diameter. Then, mice were sacrificed, and tumors obtained were used for histologic assays, biochemical determinations and to derive stable cell lines. Histologically, the obtained tumors are solid, highly compacted, and infiltrating local muscle tissue. The tumor tissue is highly vascularized. The cells are spindle-shaped, with a high number of mitoses, large irregular nuclei, and almost without nucleoli (Figure 1A,B), and necrotic areas are present (Figure 1C). To properly identify the tumors generated, an immunohistochemical study was carried out, showing that these tumors are positive for vimentin, smooth muscle actin, and caldesmon and negative for desmin and S100 (Figure 1D–H). Moreover, the expression pattern of CD34 has almost a negative display with scarce positive cells (Figure 1I). All these results indicate that the type of sarcoma obtained in 3MC-induced carcinogenesis in mice with a C57BL/6J genetic background could be a pleomorphic sarcoma with muscle differentiation, which could be consistent with a human pathology as leiomyosarcoma.

**Figure 2:**
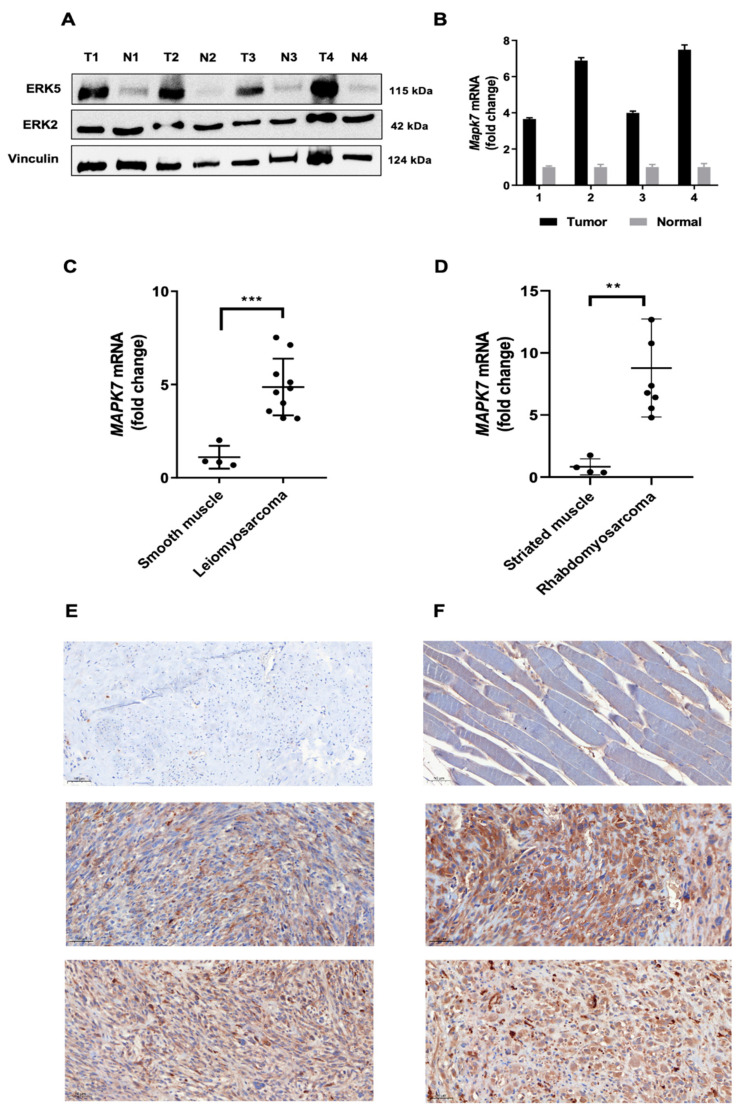
**ERK5 levels are increased in 3MC-derived tumors and in human leiomyosarcomas and rhabdomyosarcomas.** (**A**) Lysates from tumors (T) induced by 3MC and from control (N) normal muscles (n = 4) were collected, and protein extracts (100 µg) were blotted against ERK5 and ERK2 MAPKs. Vinculin was used as a loading control. (**B**) RT-qPCR analysis of *Mapk7* mRNA in the same four murine tumors induced by 3MC and control normal muscles. The mRNA expression of *Mapk1* (ERK2) was used as an endogenous control. (**C**) RT-qPCR analysis of *MAPK7* mRNA in human leiomyosarcomas and healthy smooth muscle samples. *GAPDH* was used as an endogenous control. (**D**) RT-qPCR analysis of *MAPK7* mRNA in human rhabdomyosarcomas and healthy striated muscle samples, with *GAPDH* as an endogenous control. (**E**) Representative images of immunohistochemical ERK5 staining in fixed sections of one control smooth muscle and two human leiomyosarcomas. (**F**) Representative images of immunohistochemical ERK5 staining in fixed sections of one control striated muscle and two human rhabdomyosarcomas. Images are depicted at a 20× magnification. Original blots could be found in Figure S7. ** *p* < 0.01; and *** *p* < 0.001.

To characterize the MAPK signaling in 3MC-induced sarcomas, we initially analyzed in silico the expression patterns of different MAPKs in the TGCA series, showing how sarcoma is the pathology with the highest level of ERK5 (*MAPK7*) expression, while no differences for other MAPKs were observed (Supplementary Figure S1). To extrapolate this in silico observation to our model, we performed a western blotting assay of ERK5 comparing 3MC-induced tumors with normal muscle in each case. As it is shown in Figure 2A, a marked increase in ERK5 expression was observed in tumor samples, while ERK2, the MAPK most closely related to ERK5, showed no differences. Furthermore, this observation was confirmed by RT-qPCR evaluating *Mapk7* mRNA expression levels normalized to *Mapk1* (Figure 2B). To evaluate our observation of human pathology, a short collection of human uterine leiomyosarcomas (Supplementary Table S3) was analyzed by RT-qPCR compared to smooth muscle samples. As it is shown in Figure 2C, human leiomyosarcomas showed a significant increase in the mRNA level of *MAPK7* compared to controls. In fact, immunohistochemistry was performed, showing a clear expression of this MAPK in agreement with the RT-qPCR assay (Figure 2E). Furthermore, another sarcomatous pathology of muscular origin rhabdomyosarcoma (Supplementary Table S3), was also evaluated in terms of ERK5 expression showing an even more acute expression of ERK5 compared to control striated muscle (Figure 2D,F). Therefore, this set of experiments suggests that our observations about ERK5 in the murine experimental model of 3MC-induced sarcoma can be extrapolated to human pathologies, such as leiomyosarcoma or rhabdomyosarcoma.

### 3.2. ERK5 Is a Major Determinant in the Biology of Sarcoma-Derived Cell Lines Induced by 3MC

To study the role of ERK5 in our experimental model based on 3MC, we derived cell lines from different 3MC-induced tumors. Among the several cell lines generated, we chose two of them (referred to as 3MC-C1 and 3MC-C3) that showed a potent ability to induce in vivo tumors (Supplementary Figure S2A). Indeed, tumors induced by 3MC-C1 and 3MC-C3 cell lines showed a histological pattern almost identical to the original tumors induced by 3MC (Supplementary Figure S2B). As expected, both cell lines exhibited a lack of p53 functionality (Supplementary Figure S2C), supporting previous observations in 3MC-derived tumors and also observed in human sarcomatous pathology [13,14,35,36]. Moreover, both cell lines showed a functional ERK5 signaling pathway determined by EGF stimulation (Supplementary Figure S2D).

**Figure 3:**
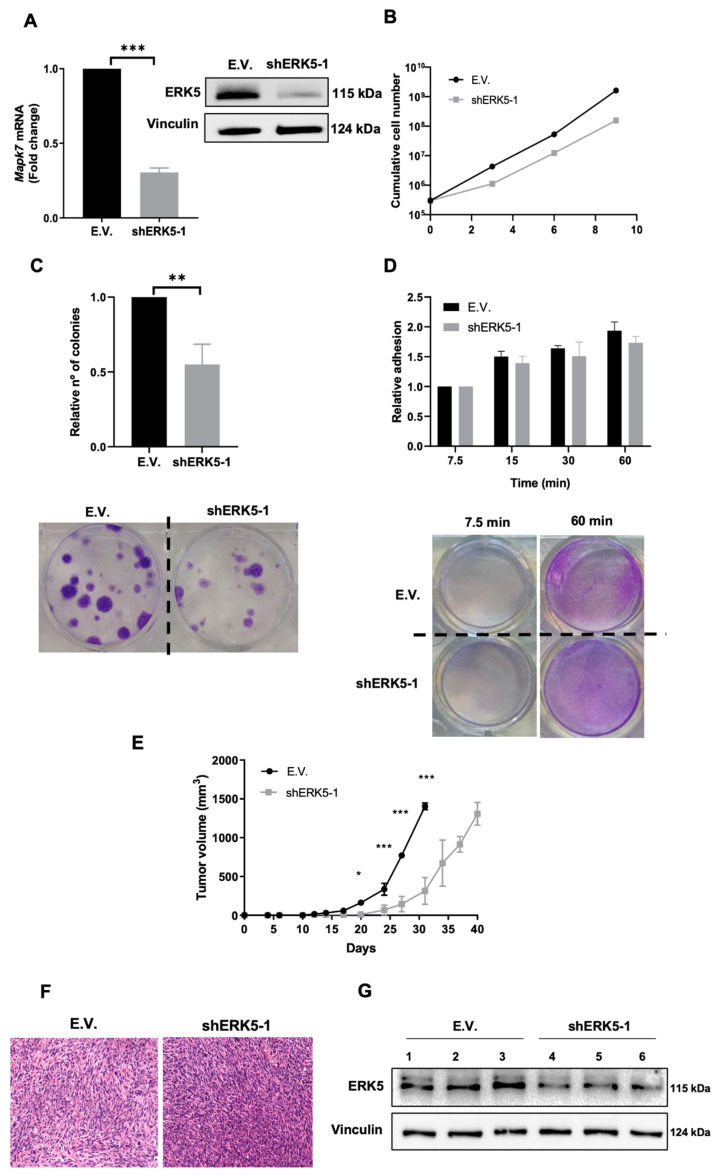
**ERK5 modulates in vitro and in vivo growth of 3MC-C1 cell line.** (**A**) 3MC-C1 cells were infected with lentiviruses carrying PLKO.1 empty vector (E.V.) or the PLKO.1-shRNA ERK5-1 vector (shERK5-1). Interference was evaluated by RT-qPCR using β-2-microglobulin *(B2m*) as an endogenous control (**left** panel). E.V. cells were considered as 1. **Right** panel shows a representative image of the interference by western blot using vinculin as a loading control. (**B**) For growth curves, 3 × 10^5^ E.V. or shERK51 3MC-C1 cells were seeded in 100 mm plates. Every 3 days, cells were counted and replated in the same manner up to day 9. Graphic shows the cumulative cell number from a representative experiment out of 3 with nearly identical results in different pools of infections. (**C**) Upper panel: Relative number of colonies obtained in clonogenic assays of E.V. and shERK5-1 3MC-C1 cells. Lower panel: Representative image of a colony formation assay from both cell lines. (**D**) Upper panel: Relative adhesion of E.V. or shERK5-1 3MC-C1 cells was assessed by crystal violet staining at the indicated time points. Lower panel: Representative image of adhesion assays at 7.5 and 60 min. (**E**) Nude mice (n = 4) were inoculated with 5 × 10^5^ cells of each cell line derived from 3MC-C1, and tumorigenesis was analyzed at indicated times. Graphics represent the mean ± SD. The experiment was performed by using another pool of infection with nearly identical results. (**F**) Western blot analysis of the expression level of ERK5 in tumors recovered from 3MC-C1 E.V. and shERK5-1. Vinculin was used as a loading control. (**G**) Representative images of the histological study of tumors obtained from 3MC-C1 derived cell lines. Pictures are shown at a 20× magnification. The graphs/histograms represent the mean ± SD of 3 independent experiments performed in triplicate cultures with different pools of infections, if not otherwise indicated. Original blots could be found in Figure S7. * *p* < 0.05; ** *p* < 0.01; and *** *p* < 0.001.

**Figure 4:**
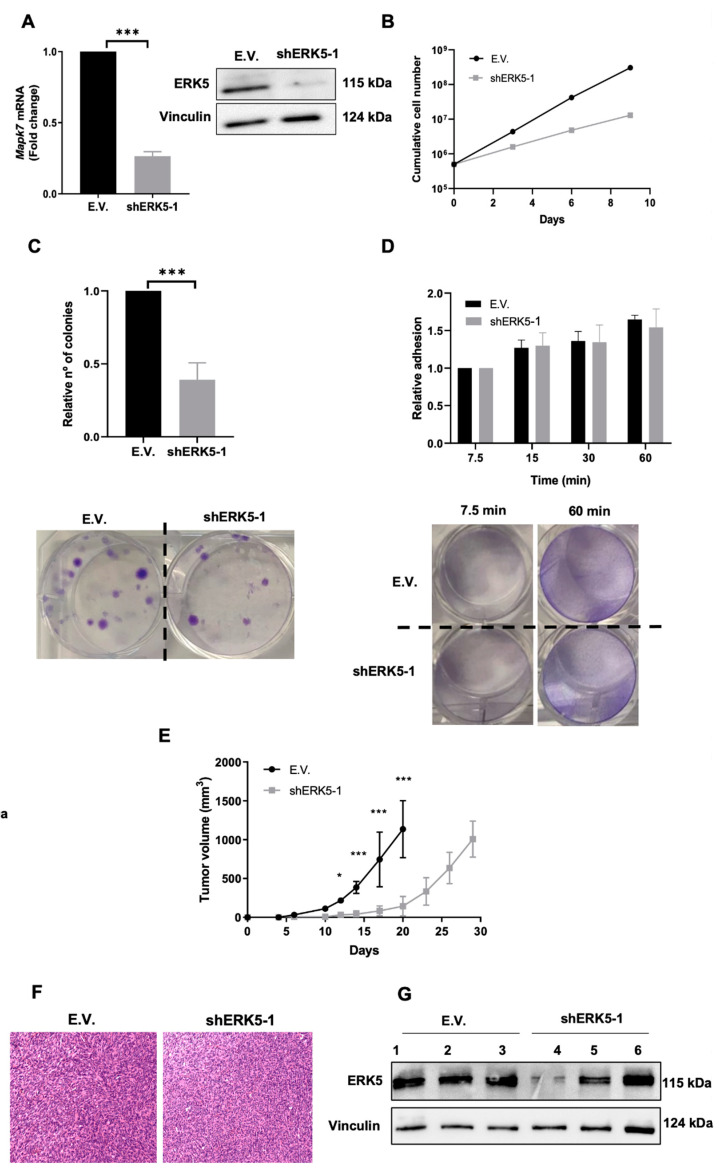
**ERK5 modulates in vitro and in vivo growth of 3MC-C3 cell line.** (**A**) 3MC-C3 cells were infected with lentiviruses carrying PLKO.1 empty vector (E.V.) or the PLKO.1-shRNA ERK5-1 vector (shERK5-1). Interference was evaluated by RT-qPCR using β-2-microglobulin *(B2m*) as an endogenous control (left panel). E.V. cells were considered as 1. Right panel shows a representative image of the interference by western blot using vinculin as a loading control. (**B**) For growth curves, 3 × 10^5^ E.V. or shERK5-1 3MC-C3 cells were seeded in 100 mm plates. Every 3 days, cells were counted and replated in the same manner up to day 9. Graphic shows the cumulative cell number from a representative experiment out of 3 with nearly identical results in different pools of infections. (**C**) Upper panel: Relative number of colonies obtained in clonogenic assays of E.V. and shERK5-1 3MC-C3 cells. Lower panel: Representative image of a colony formation assay from both cell lines. (**D**) Upper panel: Relative adhesion of E.V. or shERK5-1 3MC-C3 cells was assessed by crystal violet staining at the indicated time points. Lower panel: Representative image of adhesion assays at 7.5 and 60 min. (**E**) Nude mice (n = 4) were inoculated with 5 × 10^5^ cells of each cell line derived from 3MC-C3, and tumorigenesis was analyzed at indicated times. Graphics represent the mean ± SD. Final tumor weight for E.V.-derived tumors was 0.27 g ± 0.17 and for shERK5-1-derived tumors was 0.26 ± 0.09). The experiment was performed by using another pool of infection with nearly identical results. (**F**) Western blot analysis of the expression level of ERK5 in tumors recovered from 3MC-C1 E.V. and shERK5-1. Vinculin was used as a loading control. (**G**) Representative images of the histological study of tumors obtained from 3MC-C3-derived cell lines. Pictures are shown at a 20× magnification. The graphs/histograms represent the mean ± SD of 3 independent experiments performed in triplicate cultures with different pools of infections, if not otherwise indicated. Original blots could be found in Figure S7. * *p* < 0.05; and *** *p* < 0.001.

To fully investigate the role of ERK5 in our murine sarcoma-derived cell lines, we used specific shRNA targeting the murine *Mapk7* gene. After achieving an effective knock-down at the protein and RNA levels (Figure 3A), we evaluated the effects of ERK5 abrogated expression in terms of cell growth (Figure 3B), showing how the lack of ERK5 promoted a marked delay in cell growth, especially at later time points. Next, we decided to perform clonogenic assays to evaluate biological effects associated with ERK5 abrogation in terms of proliferation as single cells and its ability to grow as a colony. To this end, cells were seeded in 6-well plates at low density and allowed to grow for 12–14 days. Interestingly, cells with ERK5 abrogated expression exhibited a marked decrease in the number of colonies (Figure 3C), suggesting that ERK5 could have some effect not only in the clonogenic growth of our sarcoma-derived cells but also in the attachment ability, as it has been described in other experimental models [37,38]. Therefore, adhesion assays in fibronectin-coated wells were performed, but no significant differences were observed (Figure 3D). All these in vitro observations were further confirmed in the 3MC-C3 cell line (Figure 4A–D) as well as by using a second shRNA for *Mapk7* (shERK5-2) (Supplementary Figure S3A–H), which showed nearly the same results. In sum, all the evidence suggests that ERK5 signaling is directly implicated in the growth and survival abilities of our sarcoma-derived cell lines.

Next, we decided to evaluate in vivo the effect of ERK5 downmodulation. Therefore, new infection pools were obtained for both cell lines, showing an effective knock-down of ERK5 (data not shown) and injected in nude mice. As it is depicted (Figure 3E), the lack of ERK5 promoted a marked delay in the in vivo growth. In fact, when the control group was euthanized for ethical reasons on day 28 (average weight of tumors 0.32 g ± 0.9), tumors derived from the interfered lines were 5–7 times lower (Figure 3E). However, at day 39 (average weight tumors 0.31 g ± 0.13), animals inoculated with cells with abrogated ERK5 expression had developed tumors of similar size to controls, probably due to the increased proliferation rate of those cells in which the interference was less effective. In fact, analysis of tumors showed a marked recovery of ERK5 expression in tumors originating from cell lines with abrogated ERK5 expression, as well as no morphological differences between both groups (Figure 3F,G). All these observations were further confirmed in the 3MC-C3 cell line (Figure 4E–G) as well as by using a second shRNA for *Mapk7* (shERK5-2) (Supplementary Figure S3I,J), which showed nearly the same results. Taking all together, these data support the critical role of ERK5 in cell growth in vivo.

### 3.3. ERK5 Modulates Transcriptional Landscape in Sarcoma-Derived Cell Lines Induced by 3MC

**Figure 5:**
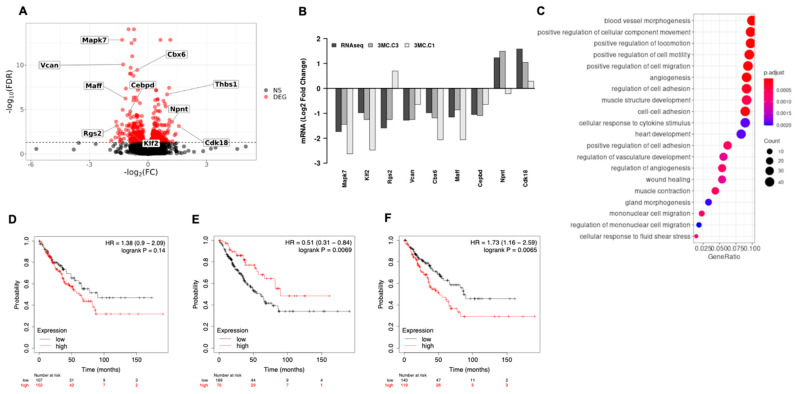
**ERK5 mediates transcriptional landscape in 3MC-derived cell lines.** (**A**) Volcano plot showing the effect size of ERK5 attenuation on gene expression (log2 fold change in ERK5-depleted over control cells) versus statistical significance of the difference in expression between conditions (−log10 of the FDR-adjusted *p*-values). Each dot represents an independent gene, and DEG (FDR < 0.05) is shown in red color. Text labels show the identity and location of the genes selected for validation. (**B**) Effect of ERK5 attenuation on the expression of the genes selected for validation. The figure shows the effect size (log2 fold change in ERK5-depleted over control cells) as determined from the RNAseq (RNAseq) data and from RT-qPCR determinations in cell pools derived from two independent infections (3MC-C3 and 3MC-C1). (**C**) Top 20 Gene Ontology terms significantly associated with genes differentially expressed upon ERK5 suppression. The graph represents the fraction of DEG with the indicated GO term. The color of the symbols indicates the statistical significance of the association (adjusted *p*-value) and their size, the absolute number of DEG genes with the indicated GO label. (**D**) Kaplan–Meyer comparing prognosis in terms of Overall Survival (OS) for two groups of patients, those with high (in red) and low (in black) expression levels of ERK5 for sarcoma patients from TCGA dataset. Hazard ratio (HR) and *p*-value (log rank P) showed in the upper right corner of the panel. (**E**) Kaplan–Meyer comparing prognosis in terms of OS for two groups of patients, those with high (in red) and low (in black) expression levels of the upregulated genes associated with ERK5 abrogation for sarcoma patients from TCGA dataset. HR and *p*-value (log rank P) showed in the upper right corner of the panel. (**F**) Kaplan–Meyer comparing prognosis in terms of OS for two groups of patients, those with high (in red) and low (in black) expression levels of the downregulated genes associated with ERK5 abrogation for sarcoma patients from TCGA dataset. HR and *p*-value (log rank P) showed in the upper right corner of the panel.

In order to identify the mechanisms by which ERK5 affects tumor growth in vivo, we determined the gene expression profile of 3MC-C1 and 3MC-C3 cells with or without ERK5 expression by means of RNA sequencing. Transcriptomic analysis showed a total of 519 differentially expressed genes (DEG) (Figure 5A) (FDR < 0.05). There were 252 genes (2.1% of the total genome) whose expression was significantly increased (LFC > 0) and 267 repressed genes (LFC < 0, 2.2% of the total genome, see Gene Expression Omnibus under the accession code GSE199395). Validation of RNAseq was performed by means of RT-qPCR by testing the expression of 9 different genes previously reported to be related to ERK5 or cancer biology (*Klf2*, *Rgs2*, *Vcan, Cbx6, Maff, Cepbd, Npnt, Cdk18*, and *Thbs1*) plus *Mapk7*, in independent pools of infection from the 3MC-C1 and 3MC-C3 cell lines (Figure 5B). Functional enrichment analysis showed several Gene Ontology biological processes that were significantly affected by ERK5 abrogation, including adhesion, migration, muscle development, etc. (Figure 5C). Interestingly, despite not being included within the top 20 significant Gene Ontology biological processes, cell proliferation (GO:0008283, 20 out of 231 genes, *p*-value 1.45× 10^−39^) and regulation of cell growth (GO:0001558, 23 out 324 *p*-value 1.57× 10^−3^), among others, were also affected. These data reinforce our previous in vitro observation of ERK5 role in cell proliferation. Next, we evaluated the potential of ERK5 expression as a biomarker for human sarcomatous pathology by performing in silico analysis in patients from the TGCA sarcoma series. As shown in Figure 5D, *MAPK7* expression does not correlate significantly with prognosis. Next, the top 30 upregulated and downregulated genes associated with ERK5 abrogation were identified, and their correlation with prognosis was explored. Interestingly, both gene expression signatures showed a statistical correlation with prognosis, finding better prognosis in terms of overall survival in patients with high levels of the genes upregulated after ERK5 abrogation (Figure 5E) and in patients with low levels of the downregulated genes (Figure 5F). These correlations suggest a link between the experimental and the clinical setup.

### 3.4. KLF2 Is a Critical Mediator of the Biological Effects of ERK5 in Sarcoma-Derived Cell Lines Induced by 3MC

**Figure 6:**
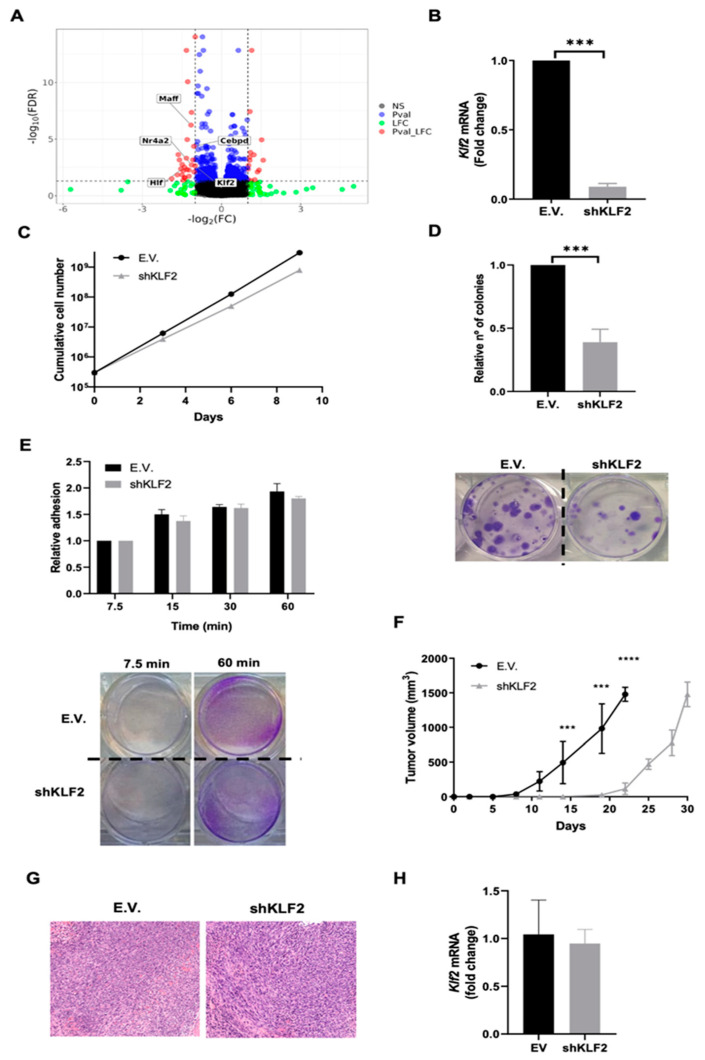
**KLF2 is a key effector of the ERK5 biological properties in 3MC-C1 cell line.** (**A**) Volcano plot showing transcription factors affected by ERK5 abrogation. Volcano plot showing the effect size of ERK5 attenuation on gene expression (log2 fold change in ERK5-depleted over control cells) versus statistical significance of the difference in expression between conditions (−log10 of the FDR-adjusted *p*-values). Each dot represents an independent gene. DEG as determined by statistical significance (FDR < 0.05), effect size (|Log2FC| > 0.99) or both are shown in blue, green, and red colors respectively. Text labels show the identity and location of the transcription factors significantly affected (FDR < 0.05 and |Log2FC| > 0.99) by ERK5. (**B**) RT-qPCR showing effective knock-down of *Klf2* expression in 3MC-C1 cells infected with lentiviral vectors coding for empty vector (E.V.) or an shRNA specific for *Klf2* (shKLF2), using β-2-microglobulin (*B2m*) as an endogenous control. E.V. cells were refereed as 1. Histogram shows the average of 3 independent pools of infection. (**C**) For growth curves, 3 × 10^5^ E.V. or shKLF2 3MC-C1 cells were seeded in 100 mm plates. Every 3 days, cells were counted and replated in the same manner up to day 9. Graphic shows the cumulative cell number from a representative experiment out of 3 with nearly identical results in different pools of infections. (**D**) Upper panel: Relative number of colonies obtained in clonogenic assays of E.V. or shKLF2 3MC-C1 cells. Colonies formed by E.V. cells were considered as 1. Lower panel: Representative image of a colony formation assay at 7.5 and 60 min. (**E**) Upper panel: Relative adhesion of E.V. or shKLF2 3MC-C1 cells was assessed by crystal violet staining at different time points. Graphic shows mean of 3 independent experiments performed in triplicated cultures with 3 different pools of infection. Lower panel: Representative image of adhesion assays at 7.5 and 60 min. (**F**) Nude mice (n = 4) were inoculated with 5 × 10^5^ E.V. and shKLF2 3MC-C1 cells, and tumor growth was analyzed at the indicated times. Final tumor weight for E.V.-derived tumors was 0.30 g ± 0.05 and for shLKF2-derived tumors was 0.29 ± 0.09. The experiment was performed by using another pool of infections with nearly identical results. (**G**) Representative images of the histological study of tumors obtained from E.V. and shKLF2 3MC-C1 cells. Pictures are depicted at a 20× magnification. (**H**) RNA from tumors induced by E.V. and shKLF2 3MC-C1 cells was extracted, and *Klf2* expression levels were measured by RT-qPCR in triplicate using *Mapk1* as an endogenous control. The graphs/histograms represent the mean ± SD of 3 independent experiments performed in triplicate cultures from different pools of infections, if not otherwise indicated. *** *p* < 0.001, **** *p* < 0.0001.

Among the genes whose expression was significantly altered (FDR < 0.05) by ERK5 knock-down, we observed 37 mouse transcription factors [39], with five of them being strongly affected (absolute log2FC ≥ 1, Figure 6A). Next, we used a protein-protein interaction network analysis based on the STRING website. This analysis indicates that only KLF2 interacts with ERK5 (Supplementary Figure S4), in agreement with previous reports showing that KLF2 is a known target of the ERK5 signaling pathway [40,41,42]. Indeed, it has been recently suggested that this transcription factor can also be a novel substrate of ERK5 kinase activity [43]. Therefore, to elucidate the role of this transcription factor in the biological effects associated with the ERK5 signaling pathway, we decided to downregulate *Klf2* expression by shRNA in 3MC-C1 cells. After achieving an effective knock-down confirmed by RT-qPCR (Figure 6B), cells were challenged in terms of proliferation, reproductive capacity, adhesion (Figure 6C–E), and in vivo growth (Figure 6F–H), obtaining the same results that in the case of ERK5 abrogation or even more striking differences. In addition, similar results were obtained in the 3MC-C3 cell line (Supplementary Figure S5). Furthermore, to fully clarify whether the effect of KLF2 could be due to a feedback regulation between KLF2 and ERK5, the RNA and protein levels and functionality of ERK5 were analyzed in control and *Klf2*-interfered cells showing no differences (Supplementary Figure S6). In sum, this set of experiments reinforces the critical role of the signaling axis ERK5→KLF2 in the sarcoma biology induced by intramuscular injection of 3MC.

## 4. Discussion

Several conclusions can be drawn from the present report. The first and most obvious is that 3MC-induced tumors are not sarcomas in a general sense as it has been previously considered [13]. Our data support that the best filiation for these tumors should be pleomorphic sarcoma with muscle differentiation that could be consistent with human leiomyosarcoma, as the histologic and immunohistochemical studies support. Therefore, our work highlights this model as an appropriate tool to study the biology of muscular sarcomas as leiomyosarcomas, both showing common genetic alterations as in the case of *Tp53* [13,44,45]. In addition, this model holds several advantages, such as the single intramuscular injection necessary to induce the tumor formation or the high effectiveness and reproducibility. However, it is remarkable that 3MC can promote rhabdomyosarcomas by subcutaneous injection in C3H/HeJ mice [46], suggesting that the use of 3MC could be a valuable tool for sarcoma research, with special interest for tumors with a muscular origin such as leiomyosarcoma or rhabdomyosarcoma.

Regarding ERK5, it is notorious how tumors induced by 3MC showed a marked upregulation of this MAPK at the protein and RNA levels, which is also observed in a human sarcomatous pathology such as leiomyosarcoma. Our data from 3MC-derived cell lines suggest that sarcoma, or at least pathologies such as leiomyosarcoma, should be included in the list of tumors in which ERK5 plays a determinant role, as for example, hepatocellular carcinoma [47], breast cancer [38,48], lung cancer [49], or myeloma [26], among others. In fact, we were able to detect a potent expression of this MAPK in a short series of rhabdomyosarcomas, indicating that further study is necessary to assess whether our observation is restricted to sarcoma of the muscular origin or can also be extrapolated to other sarcomatous pathologies. Regarding cell proliferation, although some reports suggest a lack of implication of ERK5 [50,51,52], our results obtained in growth curves and foci assays reinforce the critical role of this MAPK in cell proliferation and survival, as it has been previously described [27,49,53]. However, we cannot discard that cell type/genetic background issues, as well as different methodological approaches, could be critical to explaining the discrepancy between the different reports. Nonetheless, the oncogenic effect of ERK5 in vivo, regardless of their effects on proliferation and survival, seems to be universal and could be explained by different mechanisms. The fact that key biological processes such as motility, adhesion, blood vessel development, or angiogenesis could be affected by the lack of ERK5 fits perfectly with our observations, supporting a definitive role of ERK5 in tumor growth and progression [23]. However, some of these putative explanations can be discarded in our 3MC-based experimental model of sarcoma, for example, the effect on cell attachment described in other experimental models [38,51]. On the contrary, other possibilities, such as the role of ERK5 signaling pathway in angiogenesis [54,55,56] or even in cancer stem cell biology [57,58,59], could be considered. Therefore, further studies are necessary to fully clarify the biological mechanisms by which ERK5 is implicated in sarcomatous pathology, as could be the case of leiomyosarcomas or rhabdomyosarcomas.

Regarding KLF2, our data show that this transcription factor is a key component in the role of ERK5 signaling pathway, being able to mimic the effects associated with ERK5 in our experimental model. It is notorious how KLF2 seems to play a dual role depending on the model under study. Thus, on the one hand, there are some studies associating KLF2 with an oncogenic role, as in the case of multiple myeloma [60] or pancreatic tumors [61], whereas in other models, it seems to have a character closer to tumor suppression, as in hepatocarcinoma [62,63] or, more recently, in renal cancer [64]. Of note, *Klf2* seems to have oncogenic potential in our experimental model, being this report the first connection between this gene and sarcoma biology. Furthermore, KLF2, as an ERK5 downstream target, could be implicated in cancer through different mechanisms that remain unexplored, as could be the case of stem cell biology [65,66] or other processes such as inflammatory response [67], immune response [68], or angiogenesis [61,69]. Therefore, one possibility could be that the ERK5–KLF2 axis acts as a node in sarcoma biology by controlling several key processes.

Finally, from a clinical point of view, our murine model opens a new window of therapeutic and diagnostic possibilities for sarcomatous pathologies based on ERK5-dependent signaling [24]. In this regard, the effectivity of ERK5 inhibitors in preclinical models has been shown [49,70,71]. However, the use of ERK5 inhibitors should be carefully considered, as it has been described that they can induce the opposite effect, meaning hyperactivation of the targeted signaling pathway, as in the case of ERK1/2 [72], AKT [73], or even ERK5 [74]. From the diagnostic point of view, our in silico data suggest that ERK5 could be a weak biomarker, supporting that high levels of ERK5 may be a constitutive feature of sarcomatous pathology. However, the gene expression signature associated with ERK5 abrogation showed a strong correlation with better outcomes in the TCGA sarcoma series. This approximation, which considers the joint expression levels of 30 subrogated markers, avoids sampling effects due to the reduced size of the cohort evaluated. Therefore, the obtained results suggest that downstream effectors could be potential novel biomarkers for sarcomatous pathology. Nonetheless, the potential of ERK5 and its substrates as putative biomarkers or novel targets for therapy is a challenging issue in sarcoma biology that needs to be fully clarified.

## 5. Conclusions

In summary, the present report demonstrates a critical role for ERK5 in murine carcinogen-induced sarcoma, which correlates with an overexpression of this MAPK, also observed in human pathologies such as leiomyosarcoma or rhabdomyosarcoma. Moreover, the use of NGS allows us to conclude that ERK5 is a modulator of the transcriptional pattern in sarcoma-derived cell lines induced by 3MC and that its biological activity is mediated through the effect exerted onto KLF2. Therefore, all the previous results reveal the necessity of a more in-depth study to exploit the diagnostic and therapeutic potential of ERK5 and its downstream targets in human sarcoma.

## Data Availability

All data and materials are available on reasonable request. RNAseq analysis data for 3MC-C1 and 3MC-C3 cells with abrogated ERK5 expression have been deposited in the Gene Expression Omnibus (GEO) under the accession code GSE199395.

## Supplementary Materials

The following supporting information can be downloaded at: https://www.mdpi.com/article/10.3390/cancers14143509/s1, Figure S1: In silico analysis of RNA expression patterns of MAPK1 (ERK2), MAPK3 (ERK1), MAPK8 (JNK1), MAPK14 (p38α) and MAPK7 (ERK5) in the TGCA series, Figure S2: Characterization of cell lines from 3MC-induced tumors, Figure S3: A second shRNA for ERK5 (TRC-N-0000232397) showed identical behavior in 3MC-C1 and 3MC-C3 cell lines, Figure S4: Protein-protein interaction network, Figure S5: Abrogation of KLF2 expression in 3MC-3C cell line, Figure S6: Lack of KLF2 does not affect ERK5 expression or functionality, Figure S7: original blots; Table S1: List of antibodies used in this work, Table S2: List of RT-qPCR used in this report, Table S3: Data of patient studied, Table S4: Excel files with differential expressed genes in the RNAseq analysis.
