# Supplementary material for: ERK5 Is a Major Determinant of Chemical Sarcomagenesis: Implications in Human Pathology
Source: Cancers (Basel). 2022 Jul 19;14(14):3509. doi: 10.3390/cancers14143509 (PMC9316148; doi:10.3390/cancers14143509)
Supplement: Supplementary file 1 [file cancers-14-03509-s001.zip › cancers-1802968-supplementary.pdf]

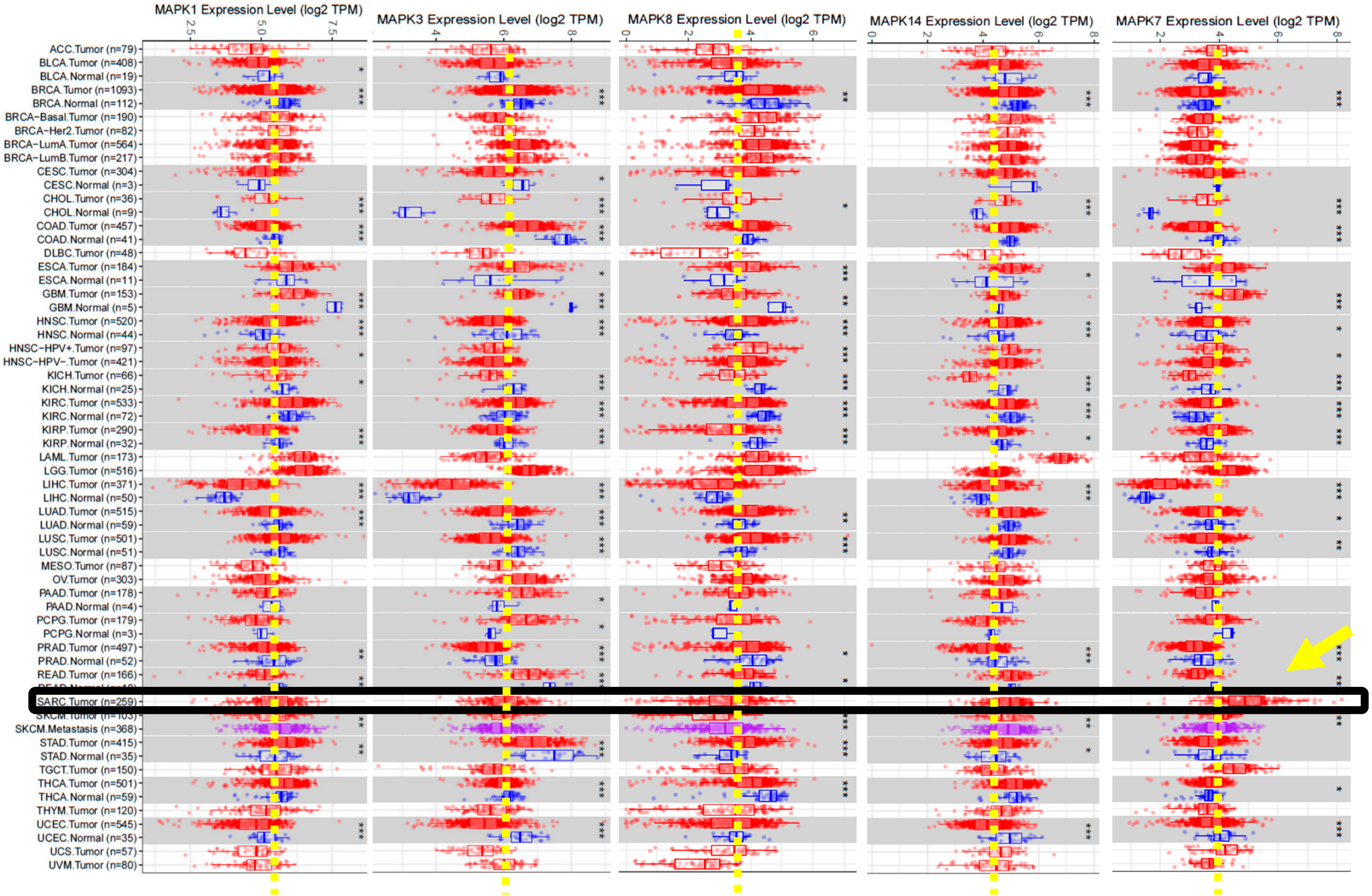

Supplementary Figure S1. ***In silico*** **analysis of RNA expression patterns of MAPK1 (ERK2), MAPK3 (ERK1), MAPK8 (JNK1), MAPK14 (p38α) and MAPK7 (ERK5) in the TGCA series**. The sarcoma series (SARC Tumor) is framed with a black rectangle. Level of mRNA *MAPK7* is indicated with a yellow arrow.

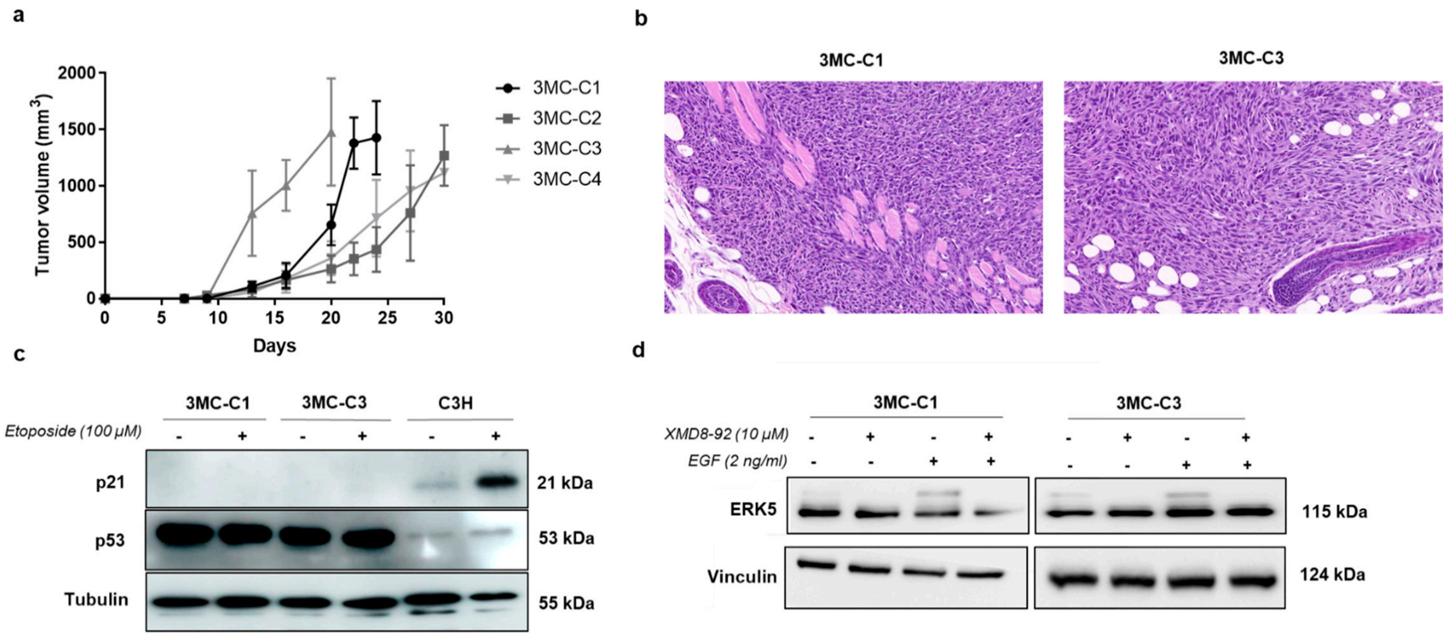

Supplementary Figure S2. Characterization of cell lines from 3MC-induced tumors. a) Nude mice (n=4) were inoculated with $5x10^5$ cells and tumor growth of parental 3MC-C1, 3MC-C2, 3MC-C3 and 3MC-C4 cell lines was monitored. **b)** Representative histological images of fixed sections of tumors obtained from 3MC-C1 and 3MC-C3 cell lines. Pictures are depicted at a 20x magnification. **c)** 3MC-C1, 3MC-C3 and CH310t1/2 (C3H) cells were treated with etoposide for 4 h. Fifty µg of protein extracts were blotted against the indicated antibodies. Tubulin was used as a loading control. **d)** 3MC-C1 and 3MC-C3 cells were incubated with XMD8-92 overnight and then treated with EGF for 15 min. Activation of ERK5 was detected by western blotting and vinculin was used as loading control. Pictures are a representative image out of 3 independent experiments. Original blots could be found in Figure S7.

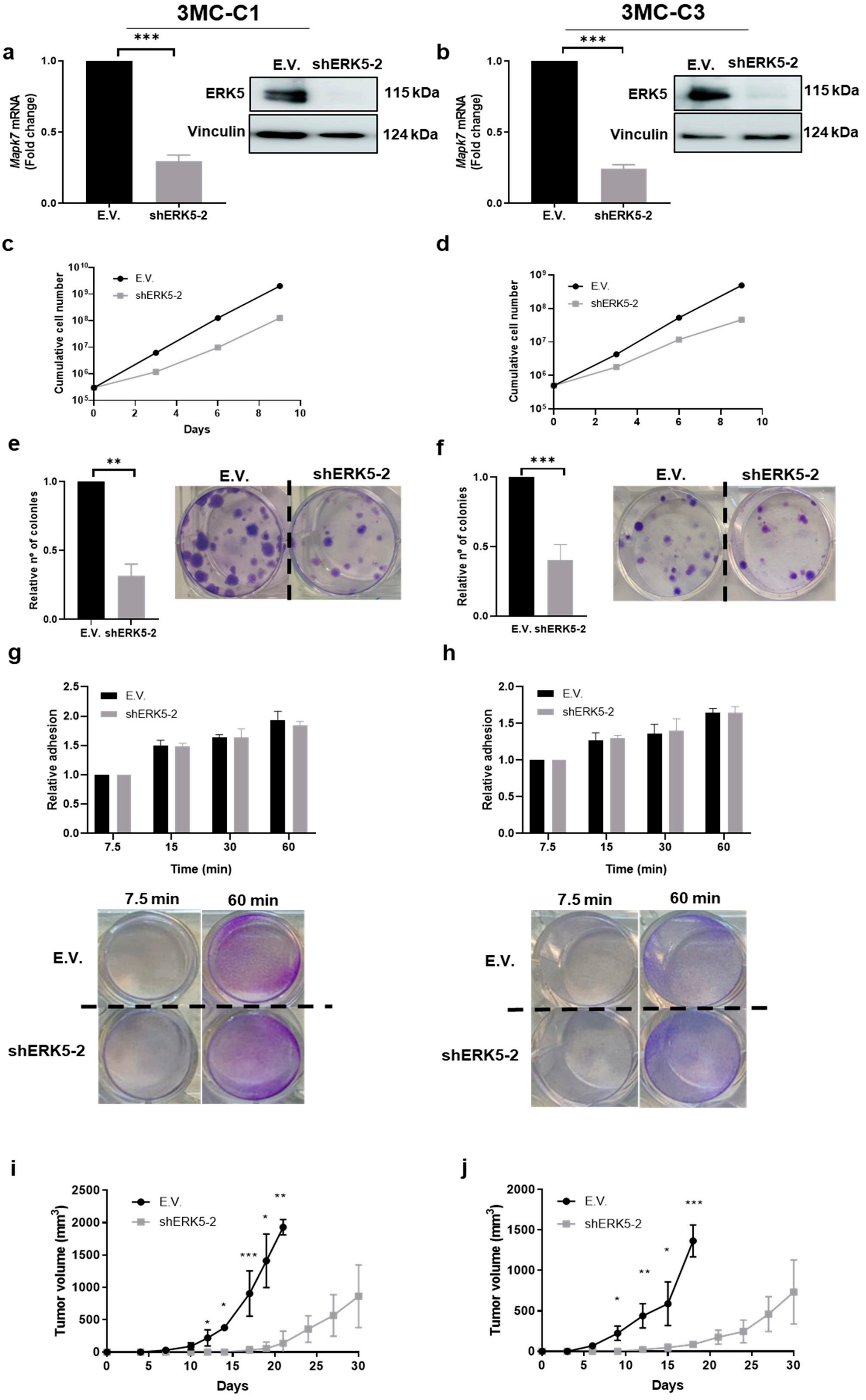
3MC-C1
3MC-C3
a
b
***
Mapk7 mRNA (Fold change)
E.V.
shERK5-2
ERK5
Vinculin
115 kDa
124 kDa
c
d
Cumulative cell number
Days
e
f
Relative nº of colonies
g
h
Relative adhesion
Time (min)
7.5 min
60 min
i
j
Tumor volume (mm3)

Supplementary Figure S3. A second shRNA for ERK5 (TRC-N-0000232397) showed identical behavior in 3MC-C1 and 3MC-C3 cell lines. 3MC-C1 **(a)** and 3MC-C3 **(b)** cells were infected with lentiviruses carrying PLKO.1 (E.V.) or a second shRNA for ERK5 (shERK5-2). Interference was evaluated by RT-qPCR using β-2-microglobulin (B2M) as an endogenous control (left panel). E.V. cells were refereed as 1. Histogram shows the average of 3 independent pools of infection. Right panel shows a representative image of the interference by western blot using vinculin as a loading control. For growth curves, $3x10^5$ E.V. or shERK5-2 3MC-C1 cells **(c)** and $5x10^5$ E.V. or shERK5-2 3MC-C3 cells **(d)** were seeded in 100 mm plates. Every 3 days, cells were counted and replated in the same manner up to day 9. Graphic shows the cumulative cells number from a representative experiment out of 3 with nearly identical results, performed with different pools of infections. Relative number of colonies obtained in clonogenic assays of 3MC-C1 **(e)** and 3MC-C3 **(f)** E.V. and shERK5-2 cells (left panel). Representative image of a colony formation assay (right panel) for 3MC-C1 and 3MC-C3. Relative adhesion of 3MC-C1 **(g)** and 3MC-C3 **(h)** E.V. or shERK5-2 cells was assessed by crystal violet staining at different time points (upper panel). Representative image of adhesion assays at 7.5 and 60 min (lower panel). 3MC-C1 **(i)** and 3MC-C3 **(j)** cell lines were infected with E.V. or shERK5-2 and nude mice (n=4) were inoculated with $5x10^5$ cells of each cell line and tumorigenesis was evaluated at the indicated times. Graphics represent the mean ± SD. The experiment was performed twice with similar results. Results represent the mean ± SD of 3 independent experiments performed in triplicate cultures with different pools of infection, if not otherwise indicated. Original blots could be found in Figure S7.

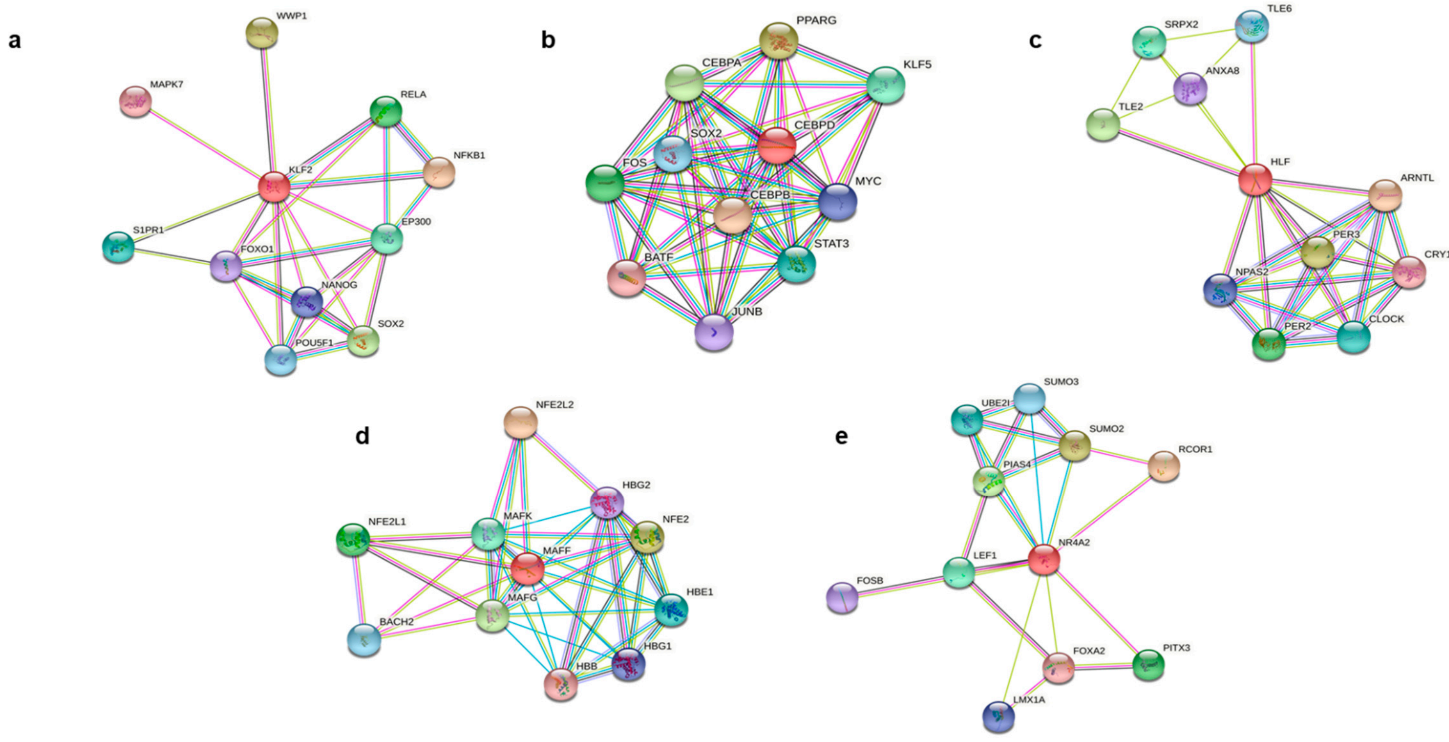

Supplementary Figure S4. Protein-protein interaction network. A Protein-protein interaction network based on the STRING web site was performed for all the transcription factors differentially regulated by ERK5: KLF2 **(a)**, CEBPB **(b)**, HLF **(c)**, MAFF **(d)**, and NR4A2 **(e)**. For each transcription factor a protein-protein interaction network query was performed. KLF2 was the unique transcription factor able to interact with ERK5.

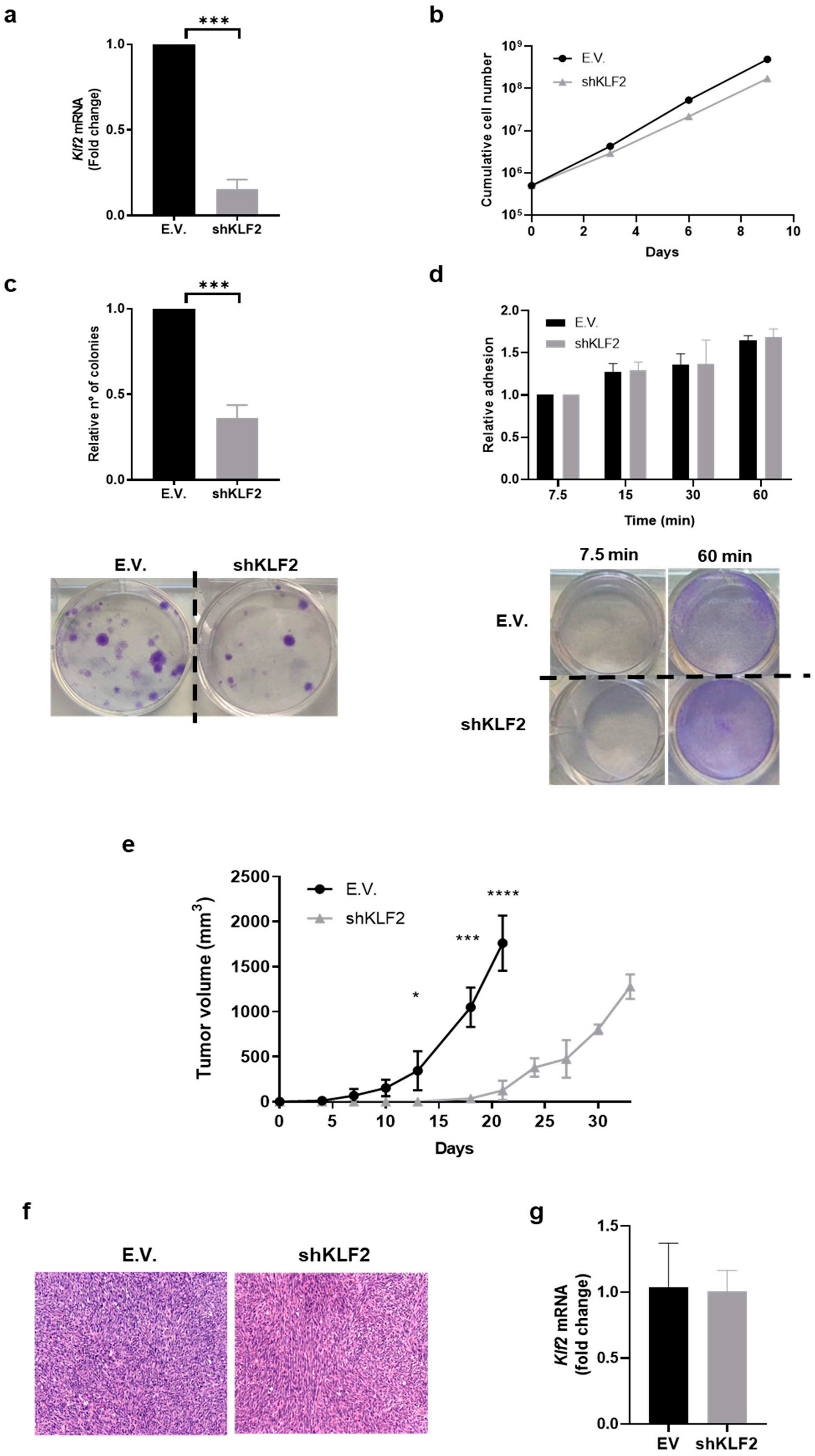
a
***
Klf2 mRNA (Fold change)
1.0
0.5
0.0
E.V.
shKLF2
b
Cumulative cell number
10^9
10^8
10^7
10^6
10^5
E.V.
shKLF2
0
2
4
6
8
10
Days
c
***
Relative n° of colonies
1.0
0.5
0.0
E.V.
shKLF2
E.V.
shKLF2
d
Relative adhesion
2.0
1.5
1.0
0.5
0.0
E.V.
shKLF2
7.5
15
30
60
Time (min)
7.5 min
60 min
E.V.
shKLF2
e
Tumor volume (mm^3)
2500
2000
1500
1000
500
0
E.V.
shKLF2
*
***
****
0
5
10
15
20
25
30
Days
f
E.V.
shKLF2
g
Klf2 mRNA (fold change)
1.5
1.0
0.5
0.0
EV
shKLF2

Supplementary Figure S5. Abrogation of KLF2 expression in 3MC-3C cell line. 3MC-C3 cells were infected with lentiviruses carrying empty vector (E.V.) or a shRNA for KLF2 (shKLF2). **a)** Interference was evaluated by RT-qPCR showing effective knockdown of *Klf2*. β-2-microglobulin (B2M) was used as an endogenous control. *Klf2* levels in E.V cells were refereed as 1. **b)** For growth curves, $5x10^5$ E.V. or shKLF2 3MC-C3 cells were seeded in 100 mm plates. Every 3 days, cells were counted and replated in the same manner up to day 9. Graphic shows the cumulative cells number from a representative experiment out of 3 with nearly identical results, from 3 different pools of infections. **c)** Upper panel: Relative number of colonies obtained in clonogenic assays of E.V. or shKLF2 3MC-C3 cells. Colonies formed by E.V. cells were considered as 1. Lower panel: Representative image of a colony formation assay. **d)** Upper panel: Relative adhesion of E.V. or shKLF2 3MC-C3 cells was assessed by crystal violet staining at different time points. Lower panel: Representative image of adhesion assays at 7.5 and 60 min. **e)** Nude mice (n=4) were inoculated with $5x10^5$ E.V. and shKLF2 3MC-C3 cells and tumor growth was analysed at the indicated times. **f)** Representative images of the histological study of tumors obtained from E.V. and shKLF2 3MC-C3 cells. Pictures are depicted at a 20x magnification. **g)** RNA from tumors induced by E.V. and shKLF2 3MC-C3 cells was extracted and *Klf2* levels measured by RT-qPCR. *Mapk1* was used as an endogenous control. Histogram/graphs represent the mean ± SD of 3 independent experiments performed in triplicate cultures with 3 different pools of infection, if not otherwise indicated.

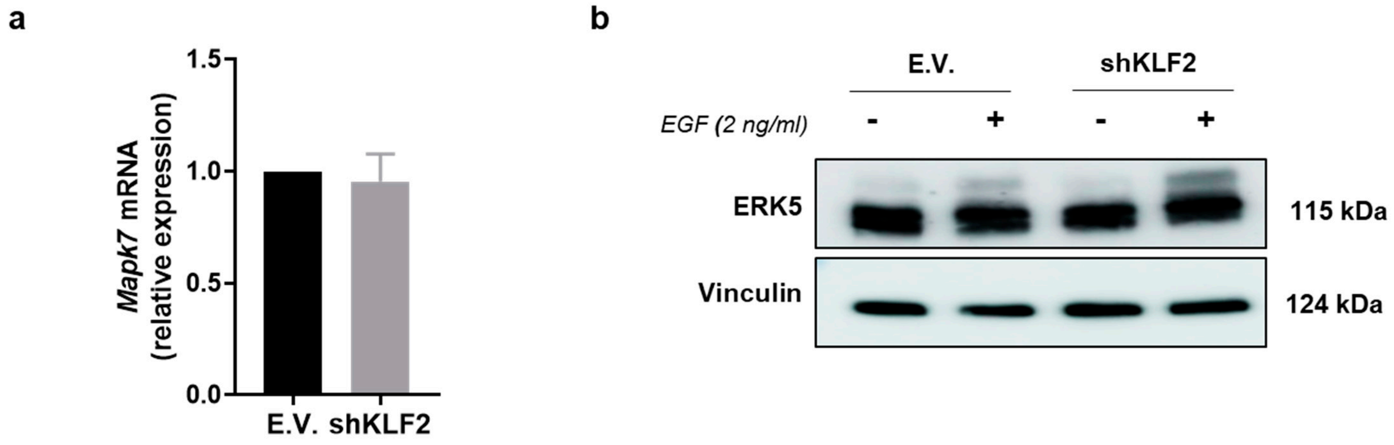

Supplementary Figure S6. Lack of KLF2 does not affect ERK5 expression or functionality. a) Levels of ERK5 were assessed by RT-qPCR in E.V. and shKLF2 3MC-C1 cells. Histogram shows the average of 3 independent pools of infection. **b)** Subconfluent E.V. and shKLF2 3MC-C1 cells were exposed to EGF at the indicated concentration for 15 min. Then, cell lysates were collected and blotted against ERK5. Vinculin was used as a loading control. Image shows a representative experiment out of 3 performed with at least 2 different pools of infections. Original blots could be found in Figure S7.

Supplementary Table S1. List of antibodies used in this work.

Antibody	Dilution	Manufacturer	Reference	Use
ERK5	1:1000	Cell Signaling Technology	3372S	Western blot
Vinculin	1:2000	Sigma-Aldrich	V9264	Western blot
Tubulin	1:500	Santa Cruz Biotechnology	DM1A	Western blot
p21	1:500	Santa Cruz Biotechnology	sc6246	Western blot
p53	1:200	Leica	P53-CM5P-L	Western blot
Anti-mouse	1:5000	Cell Signaling Technology	7076S	Western blot
Anti-rabbit	1:5000	Cell Signaling Technology	7074S	Western blot
Vimentin	1:750	Abcam	92547	Immunohistochemistry
Smooth muscle actine	1:150	Abcam	121964	Immunohistochemistry
Caldesmon	1:100	Roche	760-4375	Immunohistochemistry
Demin	1:250	Abcam	32362	Immunohistochemistry
Protein S100	1:100	Dako	IR504	Immunohistochemistry
CD34	1:100	Abcam	81289	Immunohistochemistry
ERK5	1:200	Abcam	196609	Immunohistochemistry

Supplementary Table S2. List of RT-qPCR primers used in this report.

Primer	Forward	Reverse
mouse *Mapk7*	AGATCTGTCTATGTGGTACTG	CTGGTACAGGAAGTATCTCAC
mouse *Mapk1*	CCGCACAAAAATAAGGTGCCA	GGCCAGAGCCTGTTCAACTT
mouse *B2m*	GGTCTTTCTGGTGCTTGTCTCA	GTTCGGCTTCCCATTCTCC
mouse *Npnt*	GCTGACGTCAAAAGCGTCAT	GCAGCGACCTCTTTTCAAGC
mouse *Rgs2*	AAGGAAGCTCCCAAAGAGATAAACA	TTCTGAGCTGTGGTGAAGCA
mouse *Cdk18*	CCATGGACATCCGCCTACC	TTTCCAGTTTCCCAAAGCCG
mouse *Klf2*	AAGAGCTCGCACCTAAAGGC	CTTTCGGTAGTGGCGGGTAA
mouse *Vcan*	AGGCGTCTACCGATGTGATG	CAGCGGCAAAGTTCAGAGTG
mouse *Thbs1*	TGACAACTGCCCCTACAACC	TCCATCGATGTCCACAGCAC
mouse *Cbx6*	CCACCCATCTCGCCCTTTTC	GGACCCTTGTCAATCACCTTCA
mouse *Maff*	GGAAGCTGTCAGGCAAAACG	ACTGTTCCCCCTGTGAAAGC
mouse *Cebpd*	AGAACCCGCGGCCTTCTAC	AATGTAGGCGCTGAAGTCGAT
mouse *Gas1*	CAACATGTTCGGCCCTCTTC	TGAGTCTAGGCCTCCTTTGC
human *MAPK7*	AGCACTTTAAACACGACAAC	TAGACAGATTTGAATTCGCC
human *GAPDH*	TCGTGGAAGGACTCATGACCA	CAGTCTTCTGGGTGGCAGTGA

Supplementary Table S3. Data of Patients Studied.

ID	Age	Sex	Diagnosis	Location
**Patient 01**	61	F	Leiomyosarcoma	Uterus
**Patient 02**	60	F	Leiomyosarcoma	Uterus
**Patient 03**	42	F	Leiomyosarcoma	Uterus
**Patient 04**	32	F	Leiomyosarcoma	Uterus
**Patient 05**	50	F	Leiomyosarcoma	Uterus
**Patient 06**	44	F	Leiomyosarcoma	Uterus
**Patient 07**	60	F	Leiomyosarcoma	Uterus
**Patient 08**	54	F	Leiomyosarcoma	Uterus
**Patient 09**	43	F	Leiomyosarcoma	Uterus
**Patient 10**	44	F	Leiomyosarcoma	Uterus
**Patient 11**	62	F	Fusocellular rhabdomyosarcoma	Maxillary
**Patient 12**	47	M	Fusocellular rhabdomyosarcoma	Leg
**Patient 13**	48	F	Pleomorphic rhabdomyosarcoma	Leg
**Patient 14**	51	M	Alveolar rhabdomyosarcoma	Head-neck
**Patient 15**	54	F	Alveolar rhabdomyosarcoma	Forearm
**Patient 16**	54	M	Pleomorphic rhabdomyosarcoma	Inguinal Node
**Patient 17**	36	F	Alveolar rhabdomyosarcoma	Parotid gland
**Patient 18**	72	M	Alveolar rhabdomyosarcoma	Urethra
