# Supplementary material for: ERK5 Is a Major Determinant of Chemical Sarcomagenesis: Implications in Human Pathology
Source: Cancers (Basel). 2022 Jul 19;14(14):3509. doi: 10.3390/cancers14143509 (PMC9316148; doi:10.3390/cancers14143509)

Western Blot Figures

Figura 2 (A)

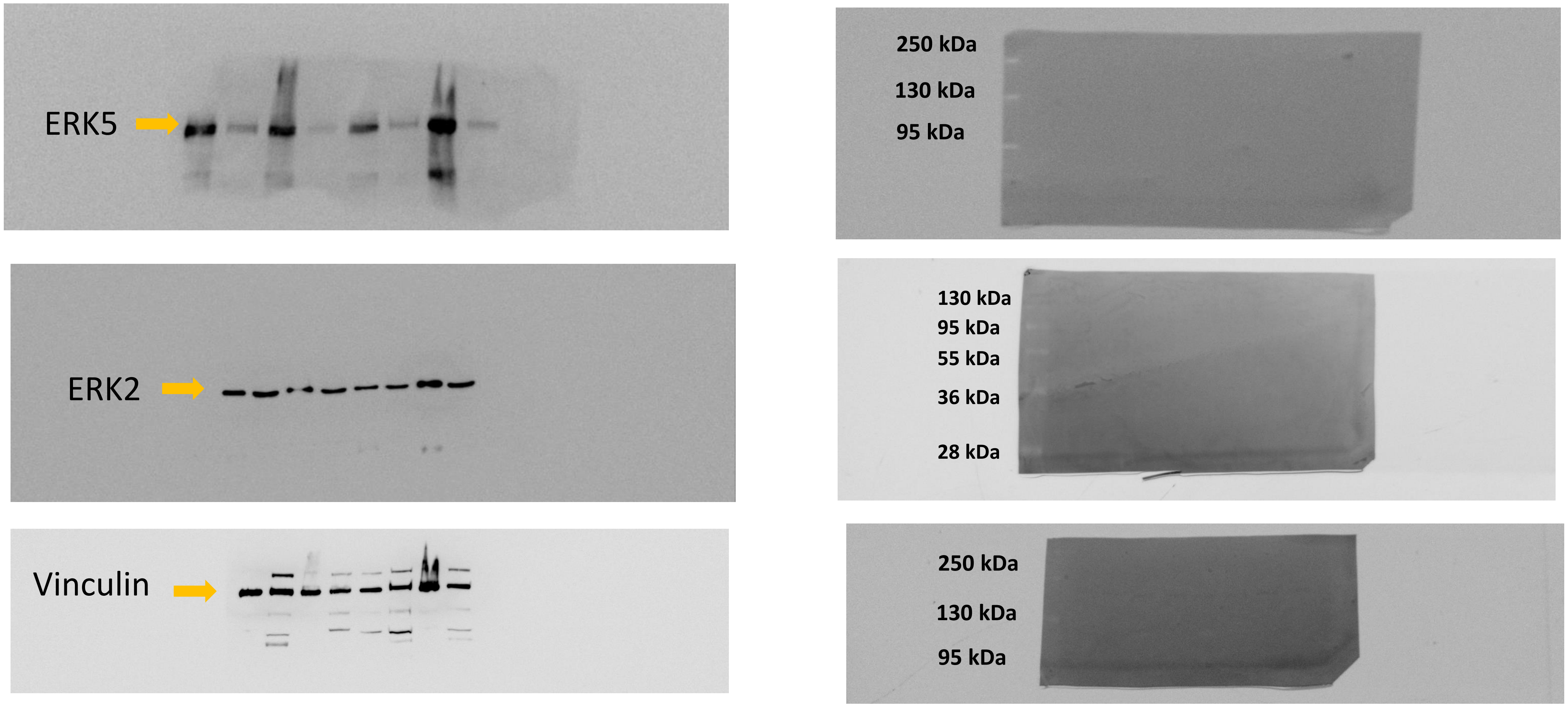
ERK5
ERK2
Vinculin
250 kDa
130 kDa
95 kDa
130 kDa
95 kDa
55 kDa
36 kDa
28 kDa
250 kDa
130 kDa
95 kDa

Figure 3 (A)

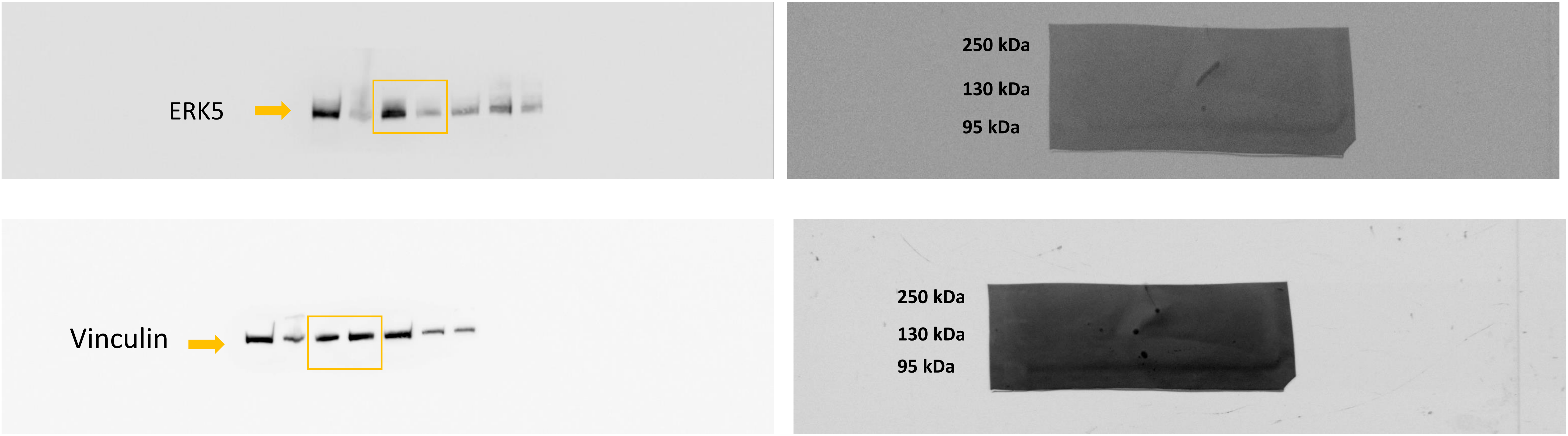

Figure 3 (G)

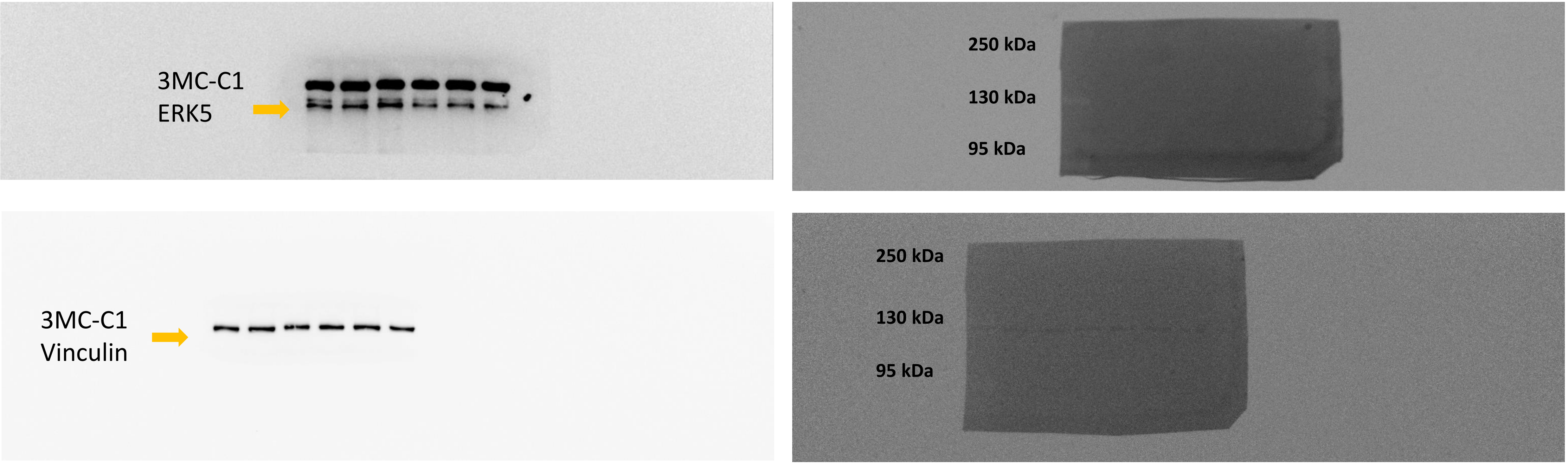
3MC-C1
ERK5
250 kDa
130 kDa
95 kDa
3MC-C1
Vinculin
250 kDa
130 kDa
95 kDa

Figure 4 (A)

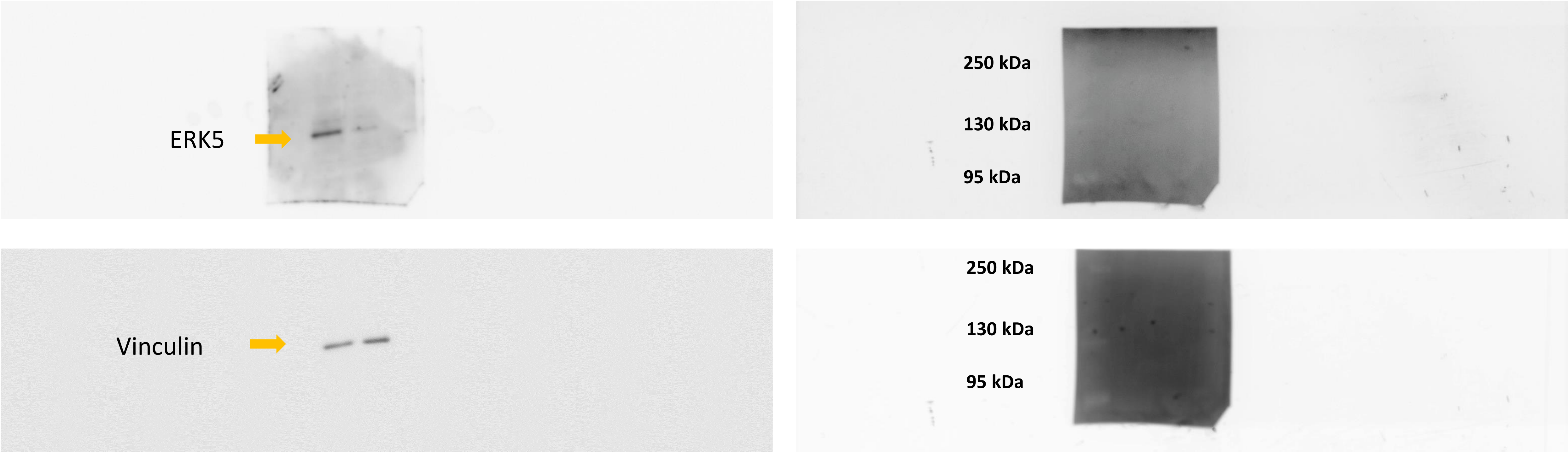

Figure 4 (G)

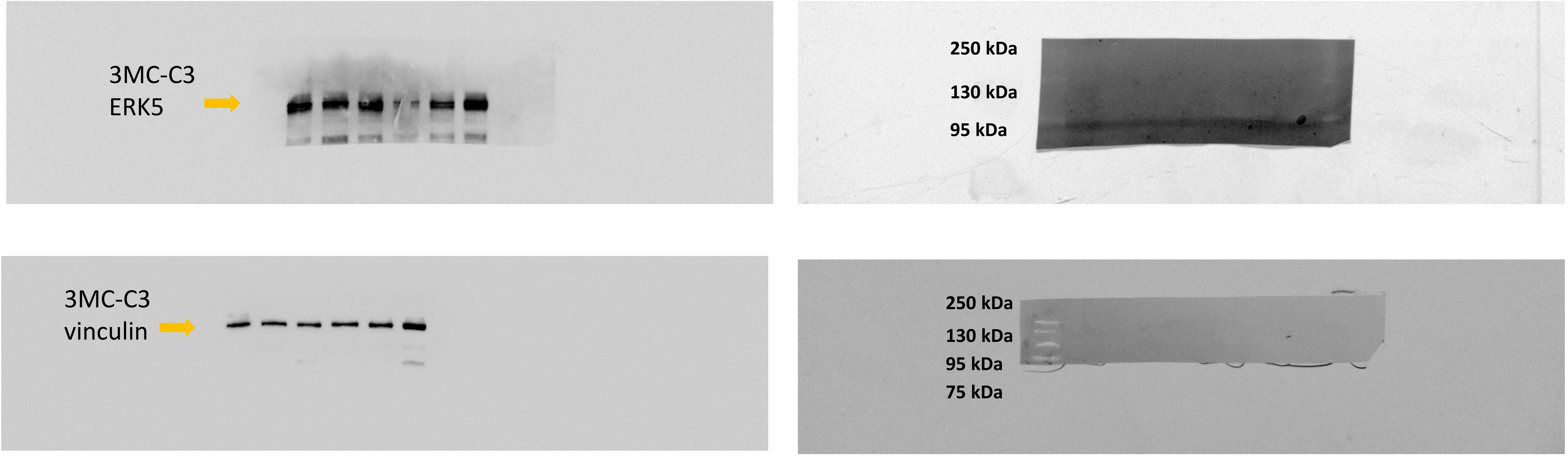
3MC-C3
ERK5
250 kDa
130 kDa
95 kDa
3MC-C3
vinculin
250 kDa
130 kDa
95 kDa
75 kDa

Supplementary Figure 2 (C)

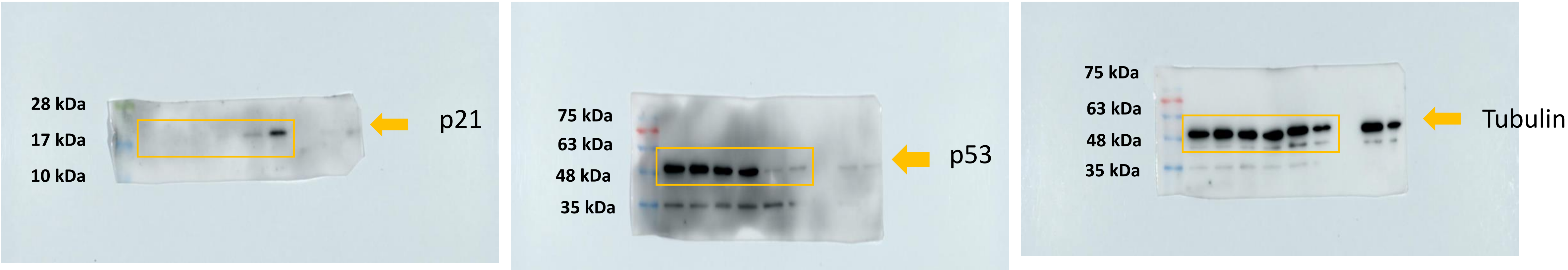

Supplementary Figure 2 (D)

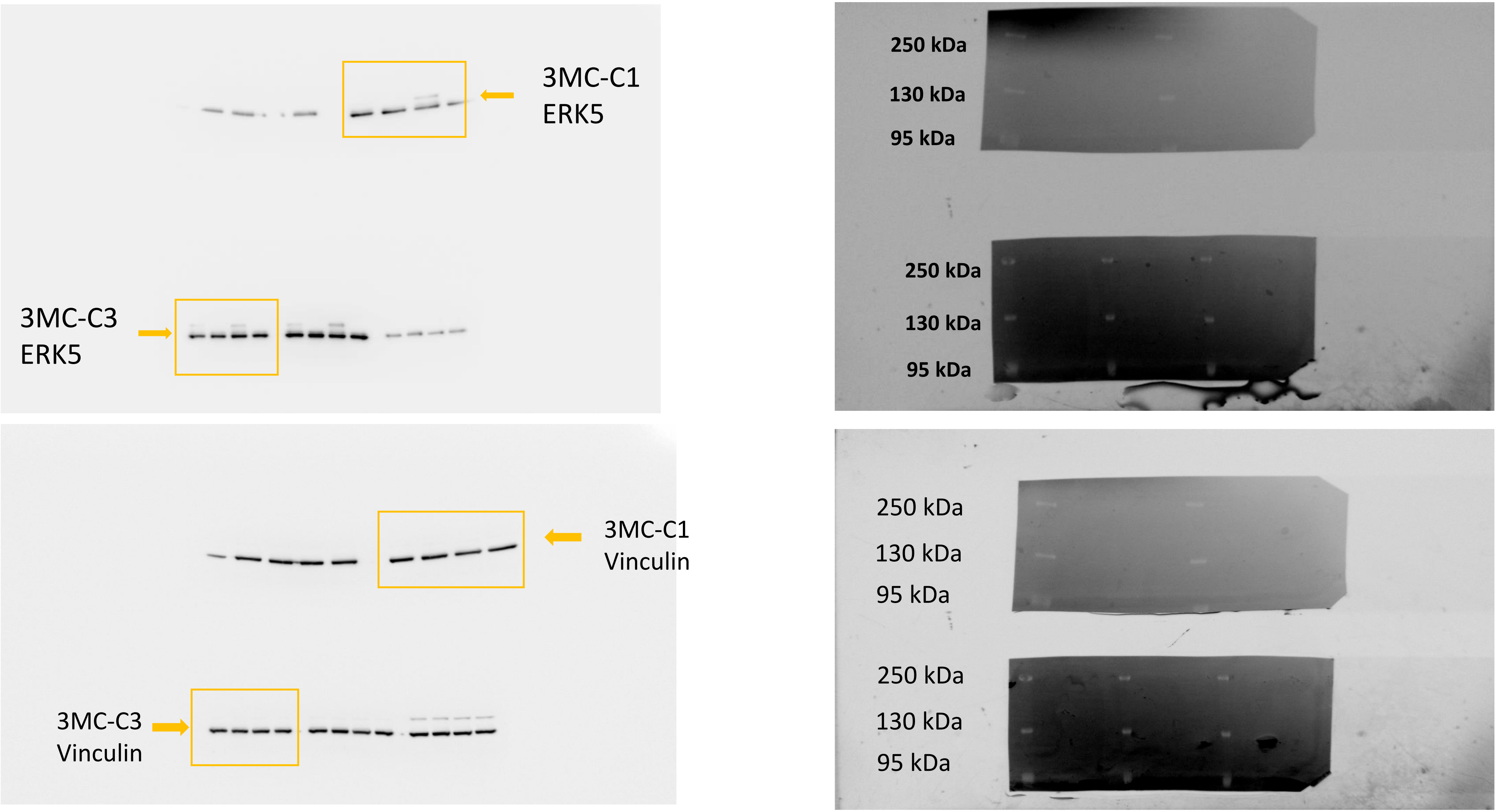

Supplementary Figure 3

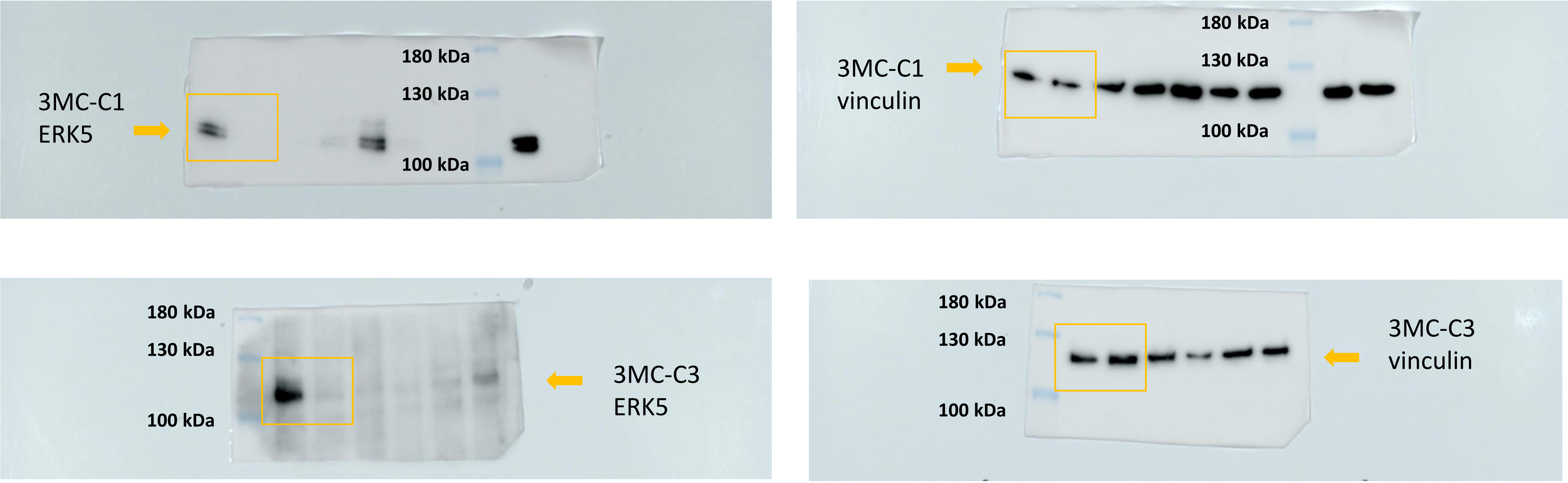

Supplementary Figure 6

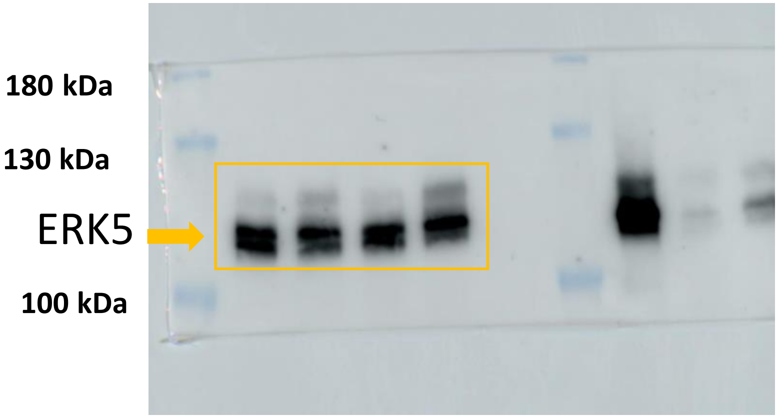

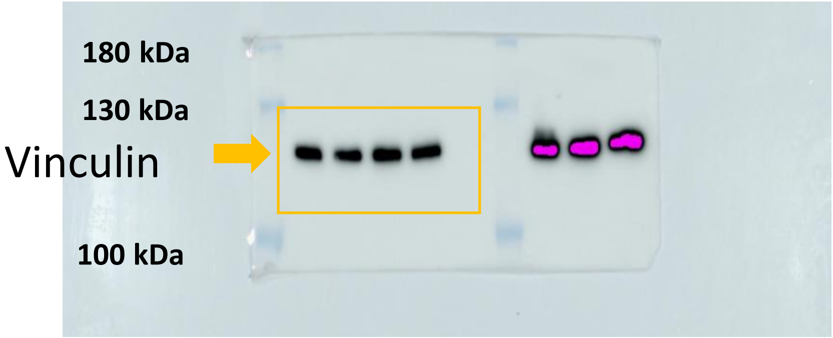

Supplement: Supplementary file 1 [file cancers-14-03509-s001.zip › Supplementary Figure S7. original blots .pdf]
